# Risk stratification of MASLD/MASH in type 2 diabetes: a pragmatic endocrinology outpatient pathway

**DOI:** 10.3389/fendo.2026.1922533

**Published:** 2026-07-30

**Authors:** Yulan Cai, Feng Zeng, Chuyu Liu

**Affiliations:** 1 Department of Endocrinology, the Second Affiliated Hospital of Zunyi Medical University, Zunyi, Guizhou, China; 2 Department of Endocrinology and Metabolism, Affiliated Hospital of Zunyi Medical University, Zunyi, Guizhou, China; 3 Thyroid and Breast Surgery, the Second Affiliated Hospital of Zunyi Medical University, Zunyi, Guizhou, China

**Keywords:** endocrinology outpatient clinic, FIB-4, fibroscan, liver fibrosis, MASH, MASLD, risk stratification, type 2 diabetes mellitus

## Abstract

Patients with type 2 diabetes mellitus (T2DM) have a high burden of metabolic dysfunction-associated steatotic liver disease (MASLD) and metabolic dysfunction-associated steatohepatitis (MASH), but liver fibrosis risk is still not assessed consistently in routine diabetes care. Recent guidelines now support active fibrosis risk stratification in metabolically high-risk populations, yet many endocrinology clinics lack a workable pathway for implementation. This narrative review proposes a risk-adapted outpatient pathway for MASLD/MASH assessment in patients with T2DM. The pathway starts with automated fibrosis-4 index (FIB-4) calculation using routine laboratory data, followed by second-line non-invasive assessment with FibroScan controlled attenuation parameter/liver stiffness measurement or enhanced liver fibrosis testing in patients with indeterminate or high-risk results. Patients at high risk should be referred for hepatology evaluation, with magnetic resonance imaging-proton density fat fraction or magnetic resonance elastography reserved for selected cases in which quantitative assessment or further staging would alter management. The review also addresses implementation barriers, appropriate use of non-invasive tests, treatment strategies with metabolic and liver-related evidence, and validation indicators for real-world assessment. By linking screening, referral, intervention, follow-up and quality control, the proposed pathway aims to move MASLD/MASH care in T2DM from incidental recognition toward structured, measurable and multidisciplinary risk management.

## Highlights

- Patients with T2DM should be recognized as a high-risk population for MASLD/MASH-related fibrosis.
- FIB-4 is a practical first-line triage tool, but should not be used as a stand-alone stopping rule.
- FibroScan CAP/LSM or ELF testing can refine risk in patients with indeterminate or high-risk FIB-4 results.
- High-risk patients require clear hepatology referral pathways and selective use of MRI-PDFF or MRE.
- A 24-week and 48-week follow-up framework can link screening, intervention, reassessment and quality control.

## Introduction

Metabolic dysfunction-associated steatotic liver disease (MASLD) has emerged as one of the most clinically relevant, yet still insufficiently recognized, chronic complications in patients with type 2 diabetes mellitus (T2DM). The recent multisociety Delphi consensus replaced the term non-alcoholic fatty liver disease (NAFLD) with MASLD, emphasizing that hepatic steatosis should be interpreted within the broader context of cardiometabolic dysfunction rather than as an isolated liver condition (1). Within this disease spectrum, metabolic dysfunction-associated steatohepatitis (MASH) represents the progressive inflammatory phenotype that may lead to clinically significant fibrosis, cirrhosis, hepatocellular carcinoma, and liver-related mortality. For patients with T2DM, MASLD/MASH is therefore not merely an incidental radiological finding, but a metabolic complication with important prognostic implications.

The disease burden of MASLD/MASH in T2DM is substantial, but only a brief orientation is needed here because the detailed evidence is summarized later in **Table 1**. Meta-analytic data indicate that NAFLD affects approximately 55.5% of individuals with T2DM and that advanced fibrosis is present in approximately 17.0% of patients with coexisting NAFLD and T2DM (2). An updated global analysis reported a pooled NAFLD/MASLD prevalence of 65.33% among patients with T2DM (3). These figures underscore the rationale for structured fibrosis risk assessment while avoiding repetition of the full epidemiological narrative in the Introduction.

**Table 1:** Representative studies on MASLD/MASH and advanced fibrosis in patients with T2DM.

Author/year	Study type	Study population	Sample size	Assessment method	Main findings	Clinical significance
Younossi et al., 2019 (2)	Systematic review and meta-analysis	Patients with T2DM from multiple regions; studies reporting NAFLD, NASH, or advanced fibrosis	49, 419 individuals with T2DM across 80 studies	Imaging, biochemical tests, and biopsy-based assessments depending on included studies	Global NAFLD prevalence in T2DM was approximately 55.5%; NASH prevalence among evaluated patients was substantial, and advanced fibrosis was estimated in approximately 17.0% of patients with coexisting NAFLD and T2DM	Established the global burden of NAFLD/NASH in T2DM and supported the need for systematic risk assessment in diabetes care
Younossi et al., 2024 (3)	Updated systematic review and meta-analysis	Patients with T2DM included in global NAFLD/MASLD epidemiological studies	2, 224, 144 patients with T2DM across 123 studies; 2, 733 biopsy-assessed patients in 12 studies	Imaging-, laboratory-, and biopsy-based definitions of NAFLD/MASLD and NASH/MASH	Global pooled prevalence of NAFLD/MASLD among patients with T2DM was approximately 65.33%, higher than earlier estimates	Confirms that MASLD affects the majority of patients with T2DM and should be incorporated into routine metabolic disease management
Lomonaco et al., 2021 (4)	Prospective cross-sectional outpatient study	Unselected patients with T2DM attending primary care or endocrinology outpatient clinics, unaware of having NAFLD	561	VCTE/FibroScan with CAP for steatosis and LSM for fibrosis; APRI and FIB-4 also assessed	Steatosis was present in approximately 70%; fibrosis defined by LSM ≥7.0 kPa in 21%; moderate fibrosis in 6%; severe fibrosis or cirrhosis in approximately 9%. Elevated AST/ALT was present only in a minority of affected patients	Demonstrated that clinically relevant liver fibrosis is common and often clinically silent in routine T2DM outpatient care
Ciardullo et al., 2021 (5)	Cross-sectional population-based study	U.S. adults with T2DM participating in NHANES 2017–2018 with reliable transient elastography data	825	Transient elastography; CAP for steatosis and LSM for fibrosis	Steatosis was present in 73.8%, advanced fibrosis in 15.4%, and cirrhosis in 7.7% of U.S. adults with T2DM	Provides population-level evidence that advanced fibrosis is common in T2DM and supports broad fibrosis risk screening beyond hepatology clinics
Ajmera et al., 2023 (6)	Prospective prevalence study	Adults aged ≥50 years with T2DM recruited for NAFLD and fibrosis assessment	524	MRI-PDFF, MRE, laboratory testing, and clinical assessment	NAFLD prevalence was 65%; advanced fibrosis was present in 14%; cirrhosis was present in 6%; obesity amplified the risk	Supports the use of systematic screening in older patients with T2DM and suggests that lower FIB-4 thresholds may be needed to preserve sensitivity in this population
Castera et al., 2023 (7)	Prospective biopsy-based multicentre study	T2DM outpatients screened in diabetology clinics and referred for hepatology work-up; liver biopsy proposed using low ALT thresholds	713 screened; 330 underwent liver biopsy	FIB-4, VCTE, liver biopsy	Despite low ALT thresholds, biopsy-confirmed NASH was present in 58% and advanced fibrosis in 38% among biopsied patients	Shows that severe liver disease can be present despite mild liver-test abnormalities and highlights the value of routinely available data to identify T2DM patients needing liver assessment
Caussy et al., 2025 (8)	Prospective multicentre study in diabetology/nutrition settings	Patients with T2DM and/or obesity with MASLD undergoing comprehensive liver assessment	654	Blood-based NITs, FIB-4, ELF, VCTE, 2D-SWE, and liver biopsy in selected high-risk patients	Intermediate/high risk of advanced fibrosis was found in 17.6%; high risk in 9.3%; the FIB-4/VCTE algorithm showed excellent diagnostic performance; ELF with adapted cut-off could serve as an alternative to VCTE	Provides direct evidence that FIB-4 followed by VCTE or ELF is feasible and diagnostically useful in diabetology settings
Vidal-Trécan et al., 2025 (9)	Large cohort comparison of screening pathways	Patients with T2DM and MASLD evaluated in the Angiosafe-T2D cohort	1, 572	FIB-4 and FibroScan-based assessment; comparison of six international guideline pathways	Major guideline pathways showed broadly comparable performance, but FIB-4 alone was suboptimal for identifying advanced fibrosis; VCTE improved risk classification	Supports the need for a two-step pathway in T2DM, using FIB-4 as an entry test followed by elastography-based refinement

APRI, aspartate aminotransferase-to-platelet ratio index; CAP, controlled attenuation parameter; ELF, enhanced liver fibrosis test; FIB-4, fibrosis-4 index; LSM, liver stiffness measurement; MASLD, metabolic dysfunction-associated steatotic liver disease; MASH, metabolic dysfunction-associated steatohepatitis; MRE, magnetic resonance elastography; MRI-PDFF, magnetic resonance imaging-proton density fat fraction; NAFLD, non-alcoholic fatty liver disease; NASH, non-alcoholic steatohepatitis; NHANES, National Health and Nutrition Examination Survey; NIT, non-invasive test; T2DM, type 2 diabetes mellitus; VCTE, vibration-controlled transient elastography.

Despite this high burden, liver risk has historically remained peripheral in routine diabetes care. Conventional diabetes management has appropriately focused on glycaemic control and the prevention of cardiovascular, renal, retinal, neurological, and foot complications. In contrast, MASLD/MASH has often been addressed only after abnormal aminotransferase levels or incidental ultrasonographic evidence of fatty liver. This reactive approach is problematic. Serum aminotransferases may remain normal in patients with clinically significant fibrosis, and conventional ultrasonography is unable to provide reliable risk stratification for fibrosis. Since fibrosis stage is the strongest determinant of liver-related outcomes in MASLD, the central question in patients with T2DM should move beyond whether hepatic steatosis is present, towards whether the patient has significant fibrosis or is at risk of progressive MASH.

Recent clinical guidelines and consensus statements have begun to reflect this shift. The 2025 American Diabetes Association consensus report highlighted MASLD as an underrecognized complication in patients with prediabetes and T2DM and recommended systematic screening for liver fibrosis in this high-risk population (10). The 2024 EASL-EASD-EASO clinical practice guidelines similarly recommended case-finding for MASLD-related fibrosis in individuals with cardiometabolic risk factors, particularly those with T2DM, obesity, abnormal liver enzymes, or imaging evidence of steatosis (11). Chinese guidelines on the prevention and treatment of MASLD have also provided structured recommendations on screening, diagnosis, treatment, monitoring, and multidisciplinary management (12). Collectively, these recommendations indicate that MASLD/MASH should no longer be considered the sole responsibility of hepatology clinics; rather, it should be incorporated into the broader framework of chronic metabolic disease management.

However, the translation of guideline recommendations into endocrinology outpatient practice remains incomplete. The fibrosis-4 index (FIB-4), calculated from age, aspartate aminotransferase, alanine aminotransferase, and platelet count, is inexpensive, readily available, and suitable for automated calculation within electronic medical records. For these reasons, it has been recommended by several guidelines as a first-line tool for liver fibrosis risk stratification (13). Nevertheless, FIB-4 has important limitations, particularly in patients with T2DM, obesity, and at the extremes of age. A strategy relying solely on FIB-4 may underestimate fibrosis risk in some patients and may leave a large intermediate-risk group without clear downstream management (14). Therefore, FIB-4 should be regarded as an entry point for risk stratification rather than a standalone diagnostic solution.

A more clinically useful approach is to embed FIB-4 within a stepwise pathway that links initial risk assessment to second-line non-invasive testing, referral decisions, metabolic intervention, and longitudinal follow-up. Vibration-controlled transient elastography, including liver stiffness measurement (LSM) and controlled attenuation parameter (CAP), provides a practical second-line assessment of fibrosis risk and hepatic steatosis in outpatient settings. Magnetic resonance imaging-proton density fat fraction (MRI-PDFF) and magnetic resonance elastography may be useful in selected high-risk or diagnostically uncertain cases, especially when quantitative assessment is needed. Importantly, such tools should not function as disconnected tests; they should be incorporated into a structured clinical pathway that determines who should be reassessed, who should be referred to hepatology, and how therapeutic responses should be monitored over time.

Emerging evidence supports the feasibility of integrating liver fibrosis assessment into diabetes care settings. Studies evaluating FIB-4 combined with vibration-controlled transient elastography in diabetology clinics have shown that this sequential approach can improve the identification of patients at risk for advanced fibrosis (8). Other models have explored the integration of liver stiffness assessment into existing diabetes complication screening programmes, such as retinal screening visits, demonstrating that liver risk assessment can be delivered within established diabetes care workflows (15). These findings suggest that endocrinology clinics are well positioned to serve as the first clinical entry point for MASLD/MASH risk detection. Nevertheless, most available studies have focused on screening performance or test feasibility, whereas relatively few have proposed a complete outpatient pathway that includes initial screening, second-line stratification, referral thresholds, treatment alignment, and follow-up validation.

Against this background, this review proposes a MASLD/MASH risk-stratification pathway for patients with T2DM in endocrinology outpatient clinics. The pathway begins with automated FIB-4 calculation from routine laboratory data, followed by FibroScan CAP/LSM or ELF testing in patients with indeterminate or high-risk results. Patients with high-risk features may require MRI-PDFF, MRE or hepatology referral, depending on local resources and clinical judgement. The pathway also incorporates lifestyle intervention, weight management, cardiometabolic risk reduction and the rational use of glucose-lowering agents with potential hepatic benefits, including sodium-glucose cotransporter 2 inhibitors and glucagon-like peptide-1 receptor agonists. Finally, we propose validation indicators that include screening coverage, second-line testing completion, high-risk case detection, referral completion, changes in CAP, LSM or MRI-PDFF, metabolic improvement, and 24-week or 48-week follow-up adherence. The broader aim is to shift MASLD/MASH care in T2DM from incidental recognition to a proactive, measurable and multidisciplinary model of hepatic risk management.

The incremental contribution of this review is therefore not the invention of a new fibrosis score or the replacement of existing society guidance. Rather, it is an implementation-oriented synthesis that adapts current recommendations to the workflow of endocrinology outpatient clinics. Compared with existing pathways, the proposed model explicitly links first-line FIB-4 triage, second-line FibroScan CAP/LSM or ELF testing, hepatology referral, metabolic intervention, 24-week and 48-week reassessment, and quality-control indicators into a closed-loop diabetes care framework.

To avoid conflating guideline recommendations with authors’ implementation choices, the pathway is framed in this review as follows: FIB-4-based first-line triage, use of VCTE/FibroScan or ELF as downstream non-invasive tests, and hepatology referral for high-risk cases are derived from current society guidance. By contrast, the exact clinic workflow, role allocation between endocrinology and hepatology, use of 24-week and 48-week checkpoints, and the proposed quality-control indicators represent pragmatic implementation choices that should be adapted to local resources and validated prospectively.

## Scope and literature selection

This narrative review was based on a targeted literature search and guideline review. PubMed/MEDLINE, Web of Science, Google Scholar and major society websites were consulted to identify evidence relevant to MASLD/MASH risk stratification in patients with T2DM. Searches were performed for publications available up to February 2026 using combinations of the terms “type 2 diabetes”, “MASLD”, “MASH”, “NAFLD”, “NASH”, “fibrosis”, “FIB-4”, “FibroScan”, “VCTE”, “CAP”, “ELF”, “MRI-PDFF”, “MRE”, “screening”, “risk stratification”, “clinical pathway”, “endocrinology clinic” and “diabetology clinic”. We prioritized major society guidelines and consensus statements, systematic reviews, prospective cohorts, population-based elastography studies, biopsy-based investigations, randomized trials and implementation studies conducted in diabetes, obesity, primary care or hepatology settings. Studies were considered most relevant when they included patients with T2DM or high-risk metabolic populations, evaluated non-invasive fibrosis assessment, reported liver-related or implementation-relevant outcomes, or informed referral and follow-up decisions. Studies focused only on unrelated liver diseases, paediatric populations, or technical imaging development without clinical applicability to T2DM outpatient care were not emphasized. Because many pivotal studies were conducted before the nomenclature transition from NAFLD/NASH to MASLD/MASH, NAFLD/NASH-era data are used as the closest available evidence base for MASLD/MASH when the underlying populations and metabolic risk profiles are clinically comparable. The aim was not to perform a formal systematic review or meta-analysis, but to translate the most relevant evidence into a clinically usable outpatient pathway for endocrinology practice.

In response to the updated guidance and related evidence, the revised search also considered recent publications on simplified European guidance summaries, holistic MASLD/MASH management, extrahepatic cancer risk, MetALD, alcohol-related liver risk stratification using ELF, and MASLD fibrosis in patients with normal ALT.

## Shared pathobiology and clinical risk of T2DM and MASLD/MASH

T2DM and MASLD/MASH share a common metabolic background in which insulin resistance plays a central role. In insulin-resistant states, adipose tissue lipolysis increases the delivery of free fatty acids to the liver, while hyperinsulinaemia and hyperglycaemia promote hepatic *de novo* lipogenesis. The consequence is intrahepatic lipid accumulation. Steatosis alone, however, does not fully explain progressive liver injury. The transition from steatosis to MASH involves mitochondrial dysfunction, oxidative stress, endoplasmic reticulum stress, inflammatory signalling, hepatocyte injury and activation of fibrogenic pathways (16, 17). MASLD is therefore better understood as a hepatic expression of systemic metabolic dysfunction than as passive fat deposition. This view also supports moving beyond aminotransferase levels and conventional imaging when the clinical question is fibrosis risk (18).

**Figure 1:**
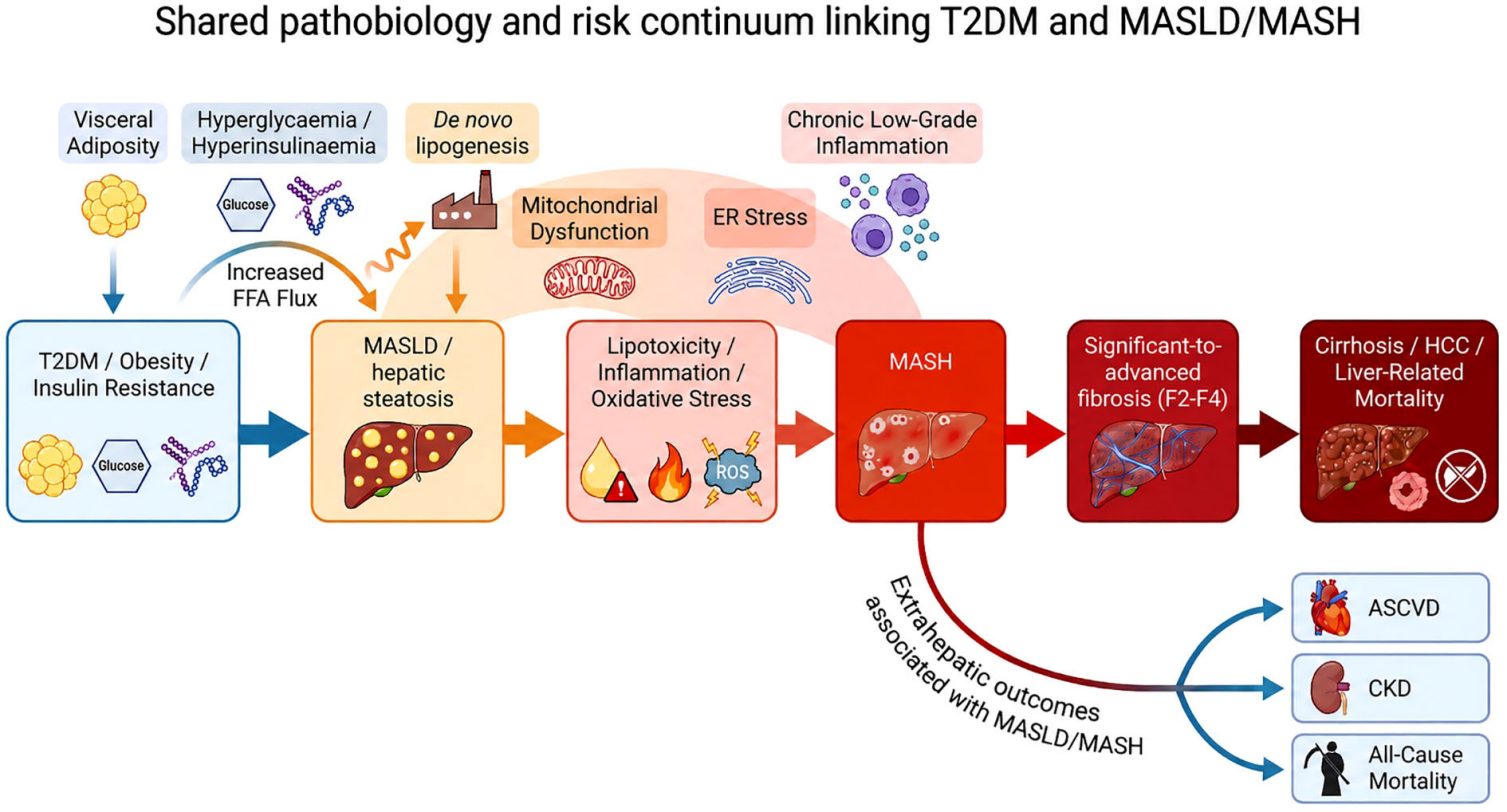
Shared pathobiology and risk continuum linking T2DM and MASLD/MASH. T2DM, obesity, visceral adiposity and insulin resistance promote hepatic steatosis through increased free fatty acid flux and *de novo* lipogenesis. Persistent lipotoxicity, mitochondrial dysfunction, endoplasmic reticulum stress, oxidative stress and chronic low-grade inflammation contribute to MASH, significant-to-advanced fibrosis, cirrhosis, hepatocellular carcinoma and liver-related mortality, while MASLD/MASH is also linked to extrahepatic outcomes including ASCVD, CKD and all-cause mortality.

The connection between T2DM and MASLD/MASH can be framed as a continuous metabolic and inflammatory cascade. Insulin resistance, visceral adiposity, hyperglycaemia and compensatory hyperinsulinaemia increase free fatty acid flux, hepatic *de novo* lipogenesis and hepatic steatosis. When hepatic lipid handling becomes maladaptive, lipotoxicity, oxidative stress, mitochondrial dysfunction, endoplasmic reticulum stress and chronic low-grade inflammation promote the transition from MASLD to MASH and progressive fibrosis. This risk continuum, together with relevant extrahepatic outcomes, is shown in **Figure 1**.

**Figure 1** illustrates why MASLD/MASH in T2DM should not be viewed simply as hepatic fat accumulation. The clinically important trajectory is the progression from metabolic stress to inflammatory liver injury, significant-to-advanced fibrosis, cirrhosis, hepatocellular carcinoma and liver-related mortality. The same metabolic environment is also linked to atherosclerotic cardiovascular disease, chronic kidney disease and all-cause mortality. This framework supports a shift from incidental detection of fatty liver to structured identification of fibrosis risk and systemic metabolic vulnerability.

Visceral adiposity further amplifies this process. Compared with subcutaneous adipose tissue, expanded visceral adipose tissue is more metabolically active and more prone to macrophage infiltration, adipokine imbalance, and increased lipolytic activity. These changes increase hepatic exposure to free fatty acids and pro-inflammatory mediators, including interleukin-6, tumour necrosis factor-α, leptin, and other signals associated with chronic low-grade inflammation. When hepatocytes are unable to safely store, oxidize, or export excessive lipid, lipotoxic species such as diacylglycerols, ceramides, saturated fatty acids, and free cholesterol accumulate and trigger cellular injury. This lipotoxic environment promotes hepatocyte ballooning, inflammatory cell recruitment, hepatic stellate cell activation, and extracellular matrix deposition, thereby connecting obesity, insulin resistance, and T2DM with progressive hepatic fibrosis (16, 17).

The association between T2DM and MASLD/MASH is bidirectional rather than merely coincidental. T2DM accelerates MASLD progression through hyperglycaemia, compensatory hyperinsulinaemia, dyslipidaemia, adipose tissue dysfunction, and systemic inflammation. Conversely, hepatic steatosis and hepatic insulin resistance can worsen glucose metabolism by increasing hepatic glucose production, impairing insulin clearance, and aggravating systemic insulin resistance. This reciprocal relationship helps explain why patients with T2DM not only have a higher prevalence of MASLD, but also have a greater probability of MASH, clinically significant fibrosis, and liver-related complications than metabolically healthier individuals with hepatic steatosis. Clinically, this means that MASLD/MASH in T2DM should be considered part of the metabolic risk continuum, rather than an incidental comorbidity.

Rather than repeating the prevalence data in detail, this section emphasizes the clinical interpretation of that burden. Across meta-analyses, elastography cohorts and biopsy-based investigations, the consistent message is that MASLD is common in T2DM and that a meaningful subgroup already has clinically relevant fibrosis or cirrhosis at assessment. Representative studies are summarized in **Table 1**.

The table is intended to provide the numerical context for the pathway rather than to duplicate the mechanistic discussion. The key implication for endocrinology care is that fibrosis risk should be assessed proactively, because liver-test abnormalities and symptoms are insufficient to identify all patients at risk.

Taken together, these studies support four practical conclusions: MASLD affects a large proportion of patients with T2DM; advanced fibrosis is not rare in routine diabetes care; aminotransferases alone are inadequate for excluding progressive liver disease; and sequential strategies that combine FIB-4 with VCTE, ELF or FibroScan-based assessment are better suited to outpatient triage than either no screening or reliance on a single test.

These conclusions justify moving from incidental recognition of steatosis to structured fibrosis risk assessment in endocrinology clinics, where patients may otherwise undergo years of metabolic follow-up without a staged liver-risk evaluation.

The clinical consequences of MASLD/MASH extend well beyond the liver. Cardiovascular disease remains one of the leading causes of morbidity and mortality in patients with MASLD, and this risk is especially important in T2DM, where baseline atherosclerotic risk is already high. A large updated meta-analysis of longitudinal studies reported that NAFLD was associated with an increased risk of fatal and non-fatal cardiovascular events, and that the risk was higher in patients with more advanced liver disease, particularly those with greater fibrosis burden (19). This observation supports the view that MASLD is not merely a marker of obesity or dysglycaemia, but may identify a subgroup of patients with more severe systemic metabolic dysfunction and higher vascular risk.

Renal risk is another clinically relevant dimension. NAFLD has been associated with an increased risk of incident chronic kidney disease, even after adjustment for conventional metabolic and cardiovascular risk factors (20). Although observational studies cannot fully establish causality, several mechanisms may plausibly link MASLD/MASH to kidney injury, including systemic inflammation, endothelial dysfunction, oxidative stress, activation of the renin–angiotensin–aldosterone system, and shared insulin-resistant pathways. For endocrinologists managing T2DM, this liver–kidney connection is important because MASLD/MASH may coexist with diabetic kidney disease and may help identify patients who require more intensive cardiometabolic and renal risk reduction.

Liver-related outcomes are largely determined by fibrosis stage. Meta-analytic evidence has shown that all-cause mortality and liver-related mortality increase progressively with advancing fibrosis in NAFLD/MASLD (21). In patients with NAFLD and T2DM, the risk is further amplified. An individual participant-level data meta-analysis demonstrated that T2DM was associated with a higher risk of hepatic decompensation and hepatocellular carcinoma among individuals with NAFLD (22). These findings are clinically important because they challenge the common perception that fatty liver in diabetes is usually benign. For a subset of patients, particularly those with advanced fibrosis, MASLD/MASH represents a progressive liver disease with measurable risks of cirrhosis, liver failure, hepatocellular carcinoma, and premature mortality.

Extrahepatic malignancy is another clinically relevant dimension of MASLD/MASH care in T2DM. Recent work on steatotic liver disease and cancer highlights biological links among metabolic dysfunction, chronic inflammation, fibrogenesis and tumorigenesis, and supports attention to both hepatic and extrahepatic cancer risks (23). In an endocrinology outpatient pathway, this should not be interpreted as a recommendation for indiscriminate cancer imaging. Rather, high-risk patients with T2DM and MASLD/MASH should receive systematic review of age-, sex- and risk-appropriate cancer screening, careful assessment for cirrhosis-related HCC surveillance when advanced fibrosis or cirrhosis is suspected, and referral to hepatology or other specialties when symptoms, abnormal tests or imaging findings suggest malignant disease.

Taken together, the shared pathobiology and adverse outcomes of T2DM and MASLD/MASH provide a strong rationale for repositioning liver fibrosis risk assessment within routine diabetes care. Endocrinology clinics are well suited for this role for several reasons. Patients with T2DM usually attend these clinics regularly; laboratory variables required for first-line fibrosis assessment, including age, aspartate aminotransferase, alanine aminotransferase, and platelet count, are commonly available; and treatment decisions related to body weight, glycaemic control, dyslipidaemia, blood pressure, and cardiometabolic protection are already made in this setting. The American Diabetes Association consensus report has emphasized the need for liver fibrosis screening and risk stratification in people with prediabetes or T2DM, particularly in the presence of obesity or other cardiometabolic risk factors (10). Similarly, the AACE clinical practice guideline, co-sponsored by the AASLD, was developed specifically for primary care and endocrinology settings and recommends FIB-4-based first-line risk assessment followed by additional non-invasive testing or imaging when appropriate (24).

Endocrinology outpatient clinics should therefore be considered a front-line setting for MASLD/MASH risk identification. This does not mean that endocrinologists should replace hepatologists in the management of advanced liver disease. Rather, endocrinology clinics can serve as the point of initial detection and triage: low-risk patients can be monitored longitudinally, intermediate-risk patients can be directed to second-line non-invasive testing, and high-risk patients can be referred for specialist hepatology assessment. This model is consistent with the broader evolution of diabetes care, in which organ complications are addressed through integrated cardiometabolic, renal and hepatic risk reduction rather than through isolated specialty pathways.

## Current guideline and consensus recommendations for MASLD/MASH screening in patients with T2DM

Recommendations for MASLD/MASH screening in patients with T2DM have changed substantially in recent years. Earlier practice often treated fatty liver as an incidental imaging diagnosis or as a hepatology issue triggered by abnormal aminotransferase levels. Current guidelines have moved toward proactive fibrosis risk stratification in patients with T2DM, because this population has a high prevalence of MASLD, a higher probability of MASH and advanced fibrosis, and increased liver-related and extrahepatic risk. Although the details differ across documents, the central message is consistent: in patients with T2DM, the first clinical task is not simply to detect steatosis, but to identify those at risk for clinically significant fibrosis.

The 2025 American Diabetes Association consensus report represents an important step in this direction. It explicitly frames MASLD as an underrecognized complication in people with prediabetes and T2DM and calls for screening for liver fibrosis and risk stratification in this high-risk population, particularly when obesity or other cardiometabolic risk factors are present (10). This recommendation is clinically meaningful because it places liver fibrosis assessment within the routine responsibilities of diabetes care. In practical terms, the ADA consensus supports the use of FIB-4 as an accessible first-line tool, followed by second-line non-invasive testing in patients with indeterminate or high-risk results. The implication for endocrinology practice is clear: liver risk should be assessed with the same seriousness as other chronic diabetes-related organ complications, rather than being deferred until liver enzymes become abnormal or cirrhosis becomes clinically apparent.

The updated AGA Clinical Care Pathway for MASLD, published in 2026, further strengthens the argument for risk-based screening in non-hepatology settings (25). The pathway is designed for broad clinical use, including primary care, diabetology, obesity medicine, and hepatology clinics. Its central logic is a stepwise approach: identify at-risk individuals, apply an initial non-invasive fibrosis score such as FIB-4, and then use second-line testing, most commonly vibration-controlled transient elastography or serum-based fibrosis tests, to refine risk and determine referral needs. Importantly, the AGA pathway recognizes that high-risk metabolic populations, including patients with T2DM, may enter fibrosis risk stratification without first requiring a separate ultrasound-based diagnosis of fatty liver. This is highly relevant to endocrinology outpatient practice, where the goal is not to perform population imaging for all patients, but to build an efficient triage system that identifies those most likely to have progressive liver disease.

The EASL-EASD-EASO 2024 Clinical Practice Guidelines similarly recommend case-finding for MASLD-related fibrosis in individuals with cardiometabolic risk factors, abnormal liver enzymes, or radiological evidence of hepatic steatosis, particularly in the presence of T2DM or obesity with additional metabolic risk factors (11). These guidelines support a sequential strategy in which simple blood-based non-invasive tests are used first, followed by elastography or other validated second-line tests in patients who require further assessment. This position is particularly important because the guideline is jointly issued by liver, diabetes, and obesity societies. It therefore reflects a multidisciplinary consensus that MASLD/MASH screening should not remain confined to hepatology clinics. For patients with T2DM, the EASL-EASD-EASO recommendations provide a strong rationale for embedding FIB-4 calculation and elastography-based risk stratification into metabolic disease management.

A simplified summary of the updated multidisciplinary European MASLD guidance has further emphasized the same practical message for non-hepatology clinicians: patients with cardiometabolic risk factors, especially those with T2DM, should be actively case-found for fibrosis using a staged non-invasive testing approach rather than managed only after overt liver disease develops (26). This simplified framing is useful for endocrinology clinics because it translates the extensive EASL-EASD-EASO guideline into a more operational sequence of risk recognition, first-line testing, downstream assessment and referral.

The 2024 Chinese guideline for the prevention and treatment of metabolic dysfunction-associated fatty liver disease provides a more regionally adapted framework for screening, diagnosis, treatment, monitoring, and follow-up (12). Its value lies not only in adopting the new MASLD terminology, but also in emphasizing practical disease management across different levels of healthcare delivery. For Chinese clinical settings, where access to FibroScan, MRI-PDFF, hepatology services, and structured metabolic clinics may vary considerably across regions and hospitals, such local guidance is particularly important. The Chinese guideline supports the use of non-invasive assessment, metabolic risk evaluation, lifestyle intervention, and long-term follow-up, all of which are essential for building a feasible endocrinology-based MASLD/MASH pathway.

The AASLD 2023 practice guidance and the AACE clinical practice guideline also provide important operational details for non-hepatology clinicians (13, 24). The AASLD guidance recommends primary risk assessment with FIB-4 in patients with suspected NAFLD/MASLD who have obesity or metabolic risk factors, followed by reassessment at appropriate intervals and further evaluation when FIB-4 is elevated or indeterminate (13). The AACE guideline, co-sponsored by the AASLD, is especially relevant to endocrinology and primary care settings. It identifies individuals with prediabetes, T2DM, obesity, metabolic syndrome, imaging-detected steatosis, or persistently elevated aminotransferases as candidates for fibrosis risk assessment, and recommends FIB-4 as the preferred initial non-invasive test (24). Together, these recommendations provide the practical basis for a clinic-level algorithm: calculate FIB-4 first, avoid unnecessary referral in clearly low-risk patients, and select patients for elastography, enhanced liver fibrosis testing, or hepatology evaluation when risk is not low.

Recent holistic care guidance for MASLD/MASH also broadens the scope of management beyond liver fibrosis alone (27). It emphasizes individualized care plans that integrate hepatic staging, cardiometabolic risk reduction, surveillance for liver-related complications, attention to extrahepatic outcomes and multidisciplinary delivery of lifestyle and pharmacological interventions. This perspective supports the present review’s view that fibrosis risk stratification should be embedded within diabetes care rather than treated as a disconnected hepatology test.

Despite differences in terminology, target populations, and preferred second-line tests, these guidelines share several key principles. First, patients with T2DM are consistently recognized as a high-risk group for MASLD/MASH and advanced fibrosis. Second, routine aminotransferase testing is not considered sufficient for excluding clinically relevant fibrosis. Third, FIB-4 is widely recommended as an initial risk stratification tool because it is inexpensive, simple, and based on routinely available clinical variables. Fourth, patients with indeterminate or high-risk FIB-4 results should undergo second-line non-invasive testing, such as vibration-controlled transient elastography, enhanced liver fibrosis testing, or other validated modalities. Fifth, patients with evidence of advanced fibrosis, cirrhosis, or diagnostic uncertainty should be referred to hepatology or managed in a multidisciplinary setting.

The differences among guidelines are equally important for clinical implementation. The ADA consensus is more explicitly diabetes-centered and frames liver fibrosis screening as a necessary part of diabetes complication management (10). The AGA pathway is more operational, with a strong emphasis on care pathways, triage, and cross-specialty applicability (25). The EASL-EASD-EASO guideline provides the broadest multidisciplinary European framework, linking liver disease management with diabetes and obesity care (11). The Chinese guideline is particularly useful for local adaptation, because it considers screening, diagnosis, treatment, monitoring, and follow-up within the Chinese healthcare context (12). The AACE guideline is highly practical for endocrinologists because it directly addresses primary care and endocrine clinical settings (24). These differences should not be viewed as contradictions. Rather, they reflect complementary perspectives that can be integrated into a pragmatic outpatient pathway.

**Figure 2:**
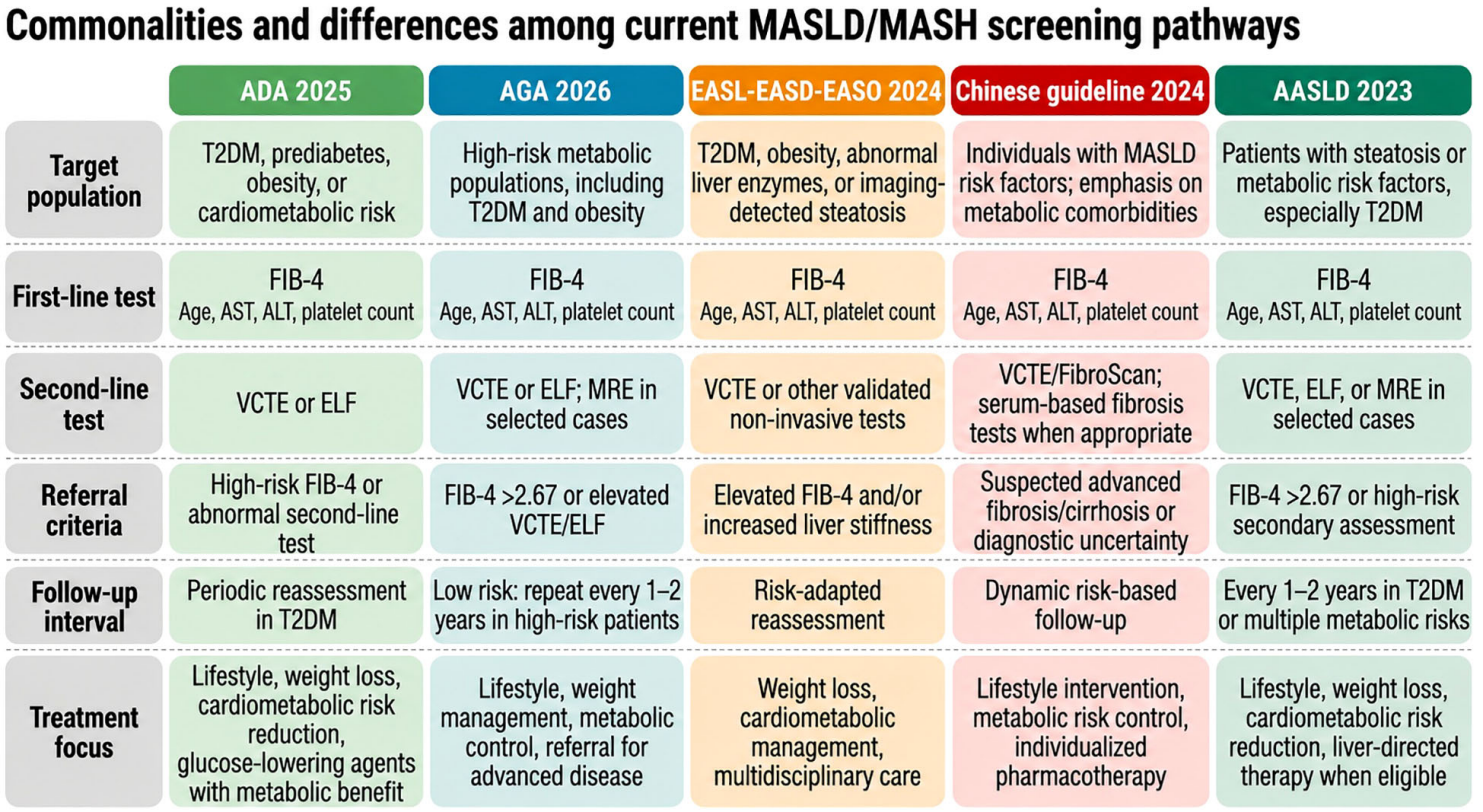
Commonalities and differences among current MASLD/MASH screening pathways. The image summarizes major recommendations from ADA 2025, AGA 2026, EASL-EASD-EASO 2024, the Chinese 2024 guideline and AASLD 2023 guidance. Across guidance documents, T2DM and metabolic risk factors define high-priority populations, FIB-4 is used as the first-line fibrosis triage tool, and VCTE/FibroScan, ELF or MRE are used for second-line assessment in selected cases. Differences remain in target populations, referral thresholds, reassessment intervals and the emphasis on metabolic, multidisciplinary and liver-directed care.

Although these guidelines differ in terminology, target populations, second-line tests, referral thresholds and follow-up intervals, they converge on a shared clinical direction. Patients with T2DM are treated as a high-risk group for MASLD/MASH-related fibrosis; FIB-4 is used as an accessible first-line triage tool; and patients with indeterminate or high-risk results are directed to second-line non-invasive testing or specialist referral. The commonalities and differences among major MASLD/MASH screening pathways are summarized in **Figure 2**.

**Figure 2** shows that the value of current guidance for endocrinology practice lies less in any isolated recommendation than in the shared operational logic across guidelines. FIB-4 is positioned as the most practical starting point because it can be calculated from routine laboratory data. VCTE/FibroScan, ELF and selected MRI-based tools are used downstream to refine risk. The main differences concern the breadth of the target population, preferred second-line tools, referral thresholds and reassessment intervals. These differences are not contradictions; they allow the pathway to be adapted to local resources, patient risk profiles and the organization of endocrinology and hepatology services.

The practical message of these guidelines is summarized in **Table 2**. Taken together, the documents support a stepwise approach in which patients with T2DM are screened for fibrosis risk using FIB-4 and those with indeterminate or high-risk results undergo second-line non-invasive testing. For endocrinology outpatient practice, the table helps translate society-level recommendations into operational decisions about who should be screened, what test should follow FIB-4, and when hepatology referral is warranted.

**Table 2:** Comparison of guidelines and consensus recommendations relevant to MASLD/MASH risk stratification in patients with T2DM.

Guideline/consensus	Year	Target population	First-line test	Second-line test	Referral recommendation	Follow-up recommendation	Implications for endocrinology outpatient clinics
ADA consensus report on MASLD in people with diabetes (10)	2025	People with prediabetes or T2DM, especially those with obesity, central adiposity, or other cardiometabolic risk factors	FIB-4 based on age, AST, ALT, and platelet count	VCTE/FibroScan or ELF; advanced imaging when clinically indicated	Refer or further evaluate patients with high-risk FIB-4 or abnormal second-line non-invasive testing	Periodic reassessment, particularly in patients with T2DM or persistent metabolic risk	Supports embedding liver fibrosis risk assessment into routine diabetes complication care rather than waiting for abnormal aminotransferases
AGA Clinical Care Pathway for MASLD (25)	2026	High-risk metabolic populations, including patients with T2DM, obesity, cardiometabolic risk factors, or imaging-detected steatosis	FIB-4 as the initial triage tool	VCTE/FibroScan or ELF; MRE or specialist evaluation in selected cases	FIB-4 >2.67, elevated VCTE/ELF, suspected advanced fibrosis, or diagnostic uncertainty should prompt hepatology evaluation	Low-risk patients may undergo repeat assessment every 1–2 years when metabolic risk remains high	Provides an operational pathway that can be adapted to diabetology clinics: FIB-4 first, second-line testing for indeterminate/high-risk patients, and referral for high-risk disease
EASL-EASD-EASO Clinical Practice Guidelines on MASLD (11)	2024	Individuals with cardiometabolic risk factors, abnormal liver enzymes, or radiological steatosis; particular emphasis on T2DM and obesity with additional metabolic risk factors	Blood-based non-invasive tests such as FIB-4	VCTE or other validated non-invasive tests for fibrosis risk refinement	Increased fibrosis risk on sequential non-invasive testing should prompt specialist assessment	Risk-adapted reassessment according to metabolic risk and fibrosis risk category	Reinforces the need for collaboration between liver, diabetes, and obesity care, supporting an endocrine-led first-step screening model with hepatology referral for high-risk patients
AASLD Practice Guidance on NAFLD/MASLD assessment and management (13)	2023	Patients with steatosis on imaging or metabolic risk factors, especially T2DM, obesity, or multiple metabolic abnormalities	FIB-4; commonly used thresholds include <1.3 for low risk and >2.67 for high risk	VCTE, ELF, or MRE in selected cases	FIB-4 ≥1.3 generally requires secondary risk assessment; FIB-4 >2.67 or high-risk secondary testing supports hepatology referral	Reassess every 1–2 years in patients with T2DM, prediabetes, or multiple metabolic risk factors; every 2–3 years in lower-risk patients	Provides practical thresholds for endocrine clinics and supports repeat fibrosis risk assessment even in initially low-risk patients with persistent metabolic risk
Chinese guideline for prevention and treatment of MASLD (12)	2024	Individuals with MASLD risk factors, including obesity, T2DM, dyslipidaemia, hypertension, metabolic syndrome, abnormal liver enzymes, or imaging-detected steatosis	FIB-4 or other validated non-invasive fibrosis scores	VCTE/FibroScan, serum-based fibrosis tests, and imaging-based assessment when appropriate	Refer patients with suspected advanced fibrosis, cirrhosis, diagnostic uncertainty, or competing liver diseases	Dynamic follow-up based on metabolic risk, liver injury, fibrosis risk, and treatment response	Provides a local framework for Chinese clinical practice and supports adaptation of FIB-4/FibroScan-based pathways to Chinese T2DM populations
AACE Clinical Practice Guideline co-sponsored by AASLD (24)	2022	Adults at high risk of NAFLD/NASH, including those with obesity, metabolic syndrome, prediabetes, T2DM, hepatic steatosis on imaging, or persistently elevated aminotransferases	FIB-4 as the preferred initial non-invasive fibrosis test	ELF or VCTE/LSM for patients with indeterminate or high FIB-4	Refer patients with indeterminate/high-risk blood tests or imaging, persistently elevated liver enzymes, hepatic steatosis plus fibrosis risk, or clinical evidence of advanced liver disease	Repeat testing every 2 years may be considered in low-risk patients; closer follow-up for indeterminate/high-risk groups	Especially relevant to endocrinology and primary care settings because it gives a practical screening-and-referral framework for non-hepatologists

ALT, alanine aminotransferase; AST, aspartate aminotransferase; AACE, American Association of Clinical Endocrinology; AASLD, American Association for the Study of Liver Diseases; ADA, American Diabetes Association; AGA, American Gastroenterological Association; EASD, European Association for the Study of Diabetes; EASL, European Association for the Study of the Liver; EASO, European Association for the Study of Obesity; ELF, enhanced liver fibrosis test; FIB-4, fibrosis-4 index; LSM, liver stiffness measurement; MASLD, metabolic dysfunction-associated steatotic liver disease; MASH, metabolic dysfunction-associated steatohepatitis; MRE, magnetic resonance elastography; T2DM, type 2 diabetes mellitus; VCTE, vibration-controlled transient elastography.

**Table 2** argues against a passive approach in which liver risk is assessed only after persistent aminotransferase elevation or incidental ultrasonographic steatosis. The shared direction is proactive fibrosis risk stratification in metabolically high-risk populations, particularly patients with T2DM. FIB-4 is a practical entry point, but it is not a diagnostic endpoint. Patients with indeterminate or high-risk results require further assessment with VCTE/FibroScan, ELF or another validated non-invasive test, and those with suspected advanced fibrosis, cirrhosis or diagnostic uncertainty should be referred to hepatology.

For endocrinology clinics, the most useful synthesis is a two- or three-step strategy. First, patients with T2DM, especially those with obesity, metabolic syndrome, abnormal liver enzymes, imaging-detected steatosis or long diabetes duration, should undergo automated FIB-4 calculation using routine data. Second, patients with indeterminate or high-risk FIB-4 values should receive FibroScan CAP/LSM or an alternative validated second-line test if elastography is unavailable. Third, patients with high liver stiffness, suspected advanced fibrosis, possible cirrhosis or discordant non-invasive results should be referred to hepatology or considered for MRI-PDFF or MRE. This approach is consistent with guideline recommendations while remaining realistic for busy diabetes clinics.

However, guideline implementation should not be reduced to a mechanical interpretation of FIB-4 thresholds. FIB-4 is useful as a first-line triage tool, but it has limitations in patients with T2DM, obesity, and at the extremes of age (14). Some patients with T2DM may still have clinically meaningful fibrosis despite a low or borderline FIB-4 result, particularly when multiple metabolic risk factors coexist. Therefore, endocrinology-based screening should combine numerical thresholds with clinical judgement. Patients with long-standing T2DM, central obesity, persistent elevation of ALT or GGT, thrombocytopenia, imaging-detected steatosis, or multiple cardiometabolic abnormalities may warrant elastography even when FIB-4 is not clearly high. Conversely, indiscriminate use of second-line testing in all patients may overload hepatology and imaging resources. The challenge is therefore to build a pathway that is both sensitive enough to avoid missing advanced fibrosis and selective enough to remain feasible.

In summary, current guidelines and consensus statements have converged on a new standard: patients with T2DM should be actively assessed for MASLD/MASH-related fibrosis risk, and this process should begin before the development of overt liver disease. The practical task now is to translate this consensus into a reproducible clinic workflow. For endocrinology outpatient practice, the most defensible model is not universal imaging, nor passive waiting for abnormal liver enzymes, but structured risk stratification beginning with FIB-4, followed by elastography or other second-line testing, hepatology referral for high-risk cases, and longitudinal reassessment as part of integrated metabolic care.

## Real-world barriers to MASLD/MASH screening in endocrinology outpatient clinics

Guidelines increasingly recommend fibrosis risk stratification in patients with T2DM, but implementation in endocrinology outpatient clinics remains uneven. The gap is not simply due to lack of evidence. It reflects barriers at the level of clinicians, patients, diagnostic resources, clinical workflow, referral systems and longitudinal disease management. Recognizing these barriers is necessary before a feasible outpatient pathway can be built.

Clinician- and system-level barriers are central. In many diabetes clinics, MASLD/MASH is still not handled as a routine diabetes-related organ complication. Surveys show persistent gaps in knowledge, screening practice and perceived responsibility across specialties (28, 29). Qualitative work in diabetes care also points to competing priorities, limited confidence in interpreting non-invasive tests and uncertainty about referral criteria as practical barriers to implementation (30).

Patient-level barriers are equally important because MASLD/MASH is usually asymptomatic until advanced disease. Public awareness remains limited, and many patients regard “fatty liver” as a benign incidental finding rather than a staged risk of fibrosis, cirrhosis, hepatocellular carcinoma and cardiovascular disease (31). Sustained lifestyle change is also difficult in the context of multimorbidity and treatment fatigue, although knowledge of fibrosis stage may improve adherence to lifestyle recommendations (32, 33).

Diagnostic and workflow barriers determine whether screening can actually proceed. FIB-4 is inexpensive, but manual calculation is unrealistic in high-volume clinics. FibroScan access may be limited or located outside endocrinology departments, and MRI-PDFF or MRE are too resource-intensive for routine first-line screening (34). Integrated pathways using automated FIB-4 and second-line testing can improve detection, but their effectiveness depends on clinician action and completion of downstream testing (35, 36).

Referral and management barriers remain a major source of lost clinical opportunity. Without explicit local thresholds, high-risk patients may be under-referred, while false-positive or low-risk cases may overload hepatology services. Current guidance supports second-line testing for indeterminate or high-risk FIB-4 and hepatology referral for suspected advanced fibrosis or cirrhosis (10, 13, 24, 25), but each institution must define who orders testing, how results are reported and when referral is mandatory.

In China, these barriers may be amplified by regional differences in diagnostic capacity and referral networks. FibroScan and MRI-PDFF are more likely to be available in tertiary hospitals than in county-level or community settings, and hepatology referral capacity may vary between provinces and hospitals. Reimbursement policies, appointment waiting times and patient travel costs can also influence whether second-line testing or specialist evaluation is completed. In regions such as Guizhou, where endocrine clinics often manage large volumes of patients with T2DM and multiple metabolic comorbidities, a staged pathway beginning with automated FIB-4 calculation may be more feasible than immediate elastography or advanced imaging for all patients.

A single educational campaign or diagnostic test will not solve these problems. A workable endocrinology pathway needs electronic FIB-4 automation, clinician education, accessible second-line testing, clear referral rules, patient-centred risk communication and predefined follow-up endpoints that connect metabolic intervention with liver-related outcomes.

## Construction of a MASLD/MASH risk-stratification pathway for patients with T2DM in endocrinology outpatient clinics

A practical MASLD/MASH pathway for endocrinology outpatient clinics should be designed around the realities of diabetes care. It must be simple enough to enter routine visits, but robust enough to identify patients with clinically relevant fibrosis. The aim is not to diagnose MASH histologically in every patient with T2DM. It is to create a reproducible triage process that separates patients suitable for longitudinal endocrine follow-up from those who need second-line non-invasive testing, advanced imaging or hepatology referral.

**Figure 3:**
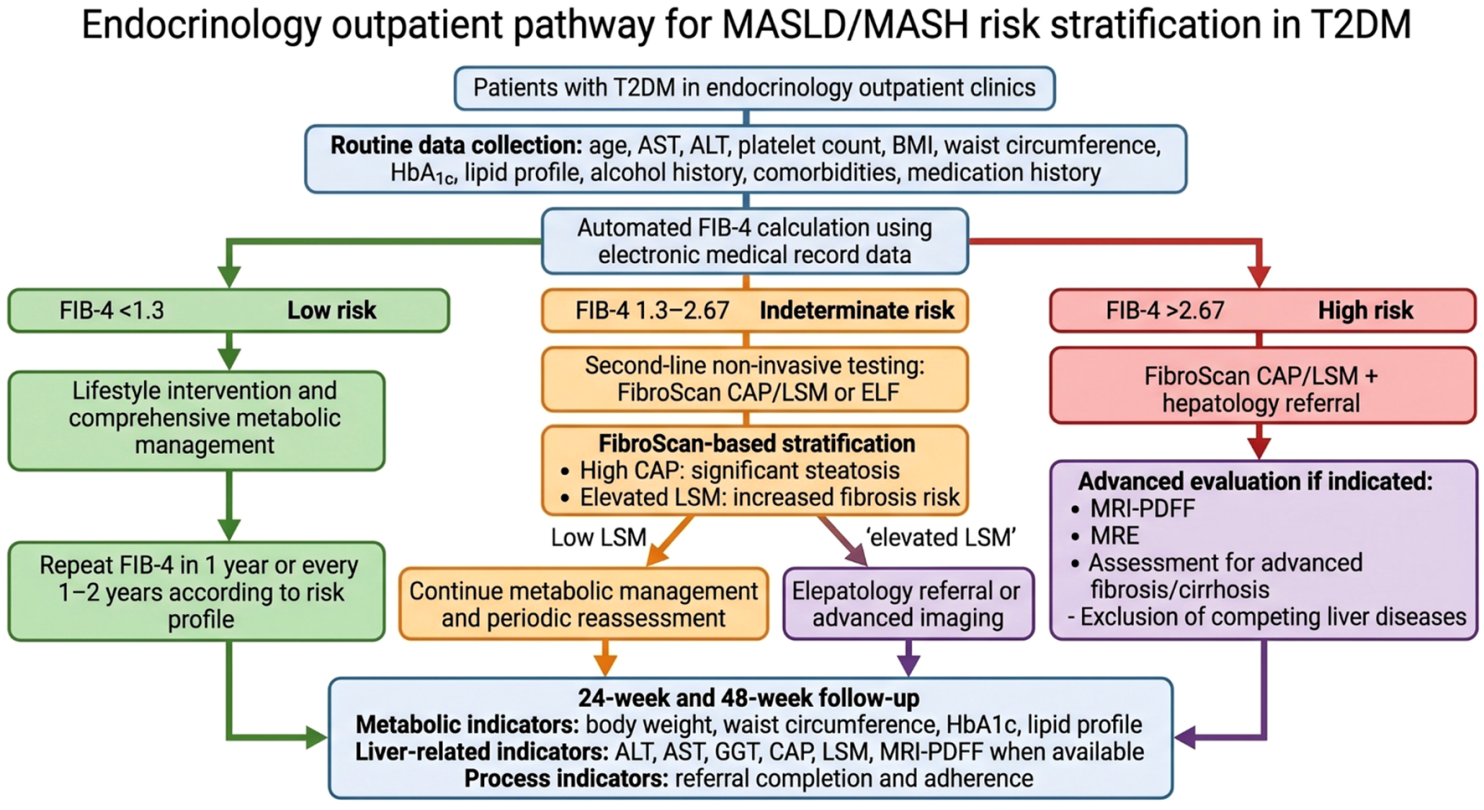
Proposed endocrinology outpatient pathway for MASLD/MASH risk stratification in patients with T2DM. Patients with T2DM undergo routine data collection and automated FIB-4 calculation, followed by low-, indeterminate- or high-risk stratification. Low-risk patients remain in endocrinology-led metabolic management with periodic reassessment; indeterminate-risk patients undergo FibroScan CAP/LSM or ELF testing; and high-risk patients require hepatology referral, with MRI-PDFF or MRE considered when clinically indicated. The FIB-4 and LSM thresholds shown in the pathway should be interpreted as pragmatic triage thresholds derived from current guidance, not as locally validated cut-offs for every population. The pathway incorporates 24-week and 48-week follow-up to assess metabolic indicators, liver-related indicators, referral completion and adherence.

To translate guideline recommendations into a clinic workflow, we propose a stepwise MASLD/MASH risk-stratification pathway for patients with T2DM. The pathway begins with routine data collection and automated FIB-4 calculation, followed by low-, indeterminate- and high-risk classification. Patients with indeterminate or high-risk results proceed to FibroScan CAP/LSM or ELF testing; those with high-risk features are considered for hepatology referral and, when appropriate, MRI-PDFF or MRE. The overall pathway is shown in **Figure 3**.

This proposed workflow should therefore be interpreted as an implementation framework built on guideline-supported components, rather than as a universally validated standard of care. The order of tests and the decision to use FibroScan CAP/LSM or ELF at specific points may reasonably differ according to local availability, payer policy, laboratory access and hepatology capacity.

**Figure 3** presents the pathway as staged triage rather than universal imaging. FIB-4 is the first-line entry point because it can be generated from routinely available laboratory data. Low-risk patients remain in endocrinology-led metabolic management with periodic reassessment. Indeterminate-risk patients undergo second-line testing with FibroScan CAP/LSM or ELF. High-risk patients require FibroScan-based assessment and hepatology referral, with MRI-PDFF or MRE reserved for selected cases in which quantitative assessment or further staging is likely to change management. The 24-week and 48-week follow-up nodes emphasize that screening must return to intervention, reassessment and adherence monitoring.

The entry population should include all patients with T2DM, with particular attention to those with obesity, central adiposity, dyslipidaemia, hypertension, elevated alanine aminotransferase or gamma-glutamyl transferase, long diabetes duration, or multiple cardiometabolic risk factors. Several guidelines now support liver fibrosis risk assessment in patients with T2DM because this population carries a high burden of MASLD, MASH, and advanced fibrosis (10, 11, 24, 25). A selective strategy limited only to patients with abnormal aminotransferases would miss a proportion of patients with clinically important fibrosis, whereas universal advanced imaging would be impractical. Therefore, the most reasonable outpatient approach is universal or near-universal first-line risk assessment using routinely available clinical and laboratory variables, followed by selective second-line testing.

Alcohol exposure should be assessed explicitly rather than treated as a peripheral exclusion criterion. Under the current steatotic liver disease nomenclature, MetALD describes patients with metabolic dysfunction and alcohol intake above MASLD thresholds but below the range usually used to define alcohol-related liver disease (37). Because MetALD shares many metabolic and fibrotic risk features with MASLD, patients with T2DM and moderate alcohol intake should not be removed from the risk-stratification workflow. Instead, alcohol history should modify triage intensity, lower the threshold for ELF, VCTE/FibroScan or hepatology referral when other risk features coexist, and prompt counselling on alcohol reduction or abstinence as part of metabolic care.

The first operational step is automated FIB-4 calculation. FIB-4 requires only age, aspartate aminotransferase, alanine aminotransferase and platelet count, which are commonly available in diabetes clinics. If clinicians must calculate the score manually, screening will remain inconsistent and dependent on individual awareness. Ideally, the electronic medical record or laboratory reporting system should generate FIB-4 automatically whenever recent liver enzyme and platelet data are available for a patient with T2DM. The result should be displayed with a risk category and a recommended next step.

FIB-4 results should then be interpreted using guideline-supported risk categories. In most adult patients, a FIB-4 value below 1.3 is commonly used to indicate low risk for advanced fibrosis, a value between 1.3 and 2.67 indicates indeterminate risk, and a value above 2.67 suggests a higher probability of advanced fibrosis and the need for further assessment or referral (13, 24, 25). These thresholds should be viewed as pragmatic starting points for triage rather than population-specific diagnostic cut-offs. In older adults, particularly those older than 65 years, higher age-adjusted thresholds may be considered to reduce false-positive results (13). Conversely, in younger adults, FIB-4 may have reduced accuracy because age is a component of the score. These caveats are particularly important in Chinese and broader Asian T2DM populations, in whom body composition, diabetes duration, obesity phenotype and access to second-line testing may differ from the cohorts in which many thresholds were derived. Thus, the pathway should be locally validated and calibrated while conventional thresholds are used provisionally to guide initial workflow.

Normal ALT should not be used as a gatekeeper for whether fibrosis assessment is needed. A recent MASLD study showed that liver fibrosis may be present despite persistently normal ALT and that this subgroup can have a distinct treatment response (38). This evidence reinforces a central operational point for diabetes clinics: patients with T2DM, obesity, imaging-detected steatosis, thrombocytopenia, long diabetes duration or clustered cardiometabolic risks should remain eligible for fibrosis risk stratification even when ALT is within the laboratory reference range.

Age-adjusted interpretation is particularly important in endocrinology clinics. Because age is a direct component of FIB-4, standard thresholds may generate false-positive results in older adults, especially those older than 65 years. McPherson and colleagues showed that applying conventional FIB-4 thresholds in older patients substantially reduced specificity and proposed a higher low-risk threshold of 2.0 for individuals older than 65 years (39). In practical terms, an older patient with a mildly elevated FIB-4 should not automatically be treated as having advanced fibrosis without confirmatory testing, whereas a younger patient with a low FIB-4 but multiple metabolic risk factors may still require clinical judgement and, when appropriate, second-line assessment.

**Figure 4:**
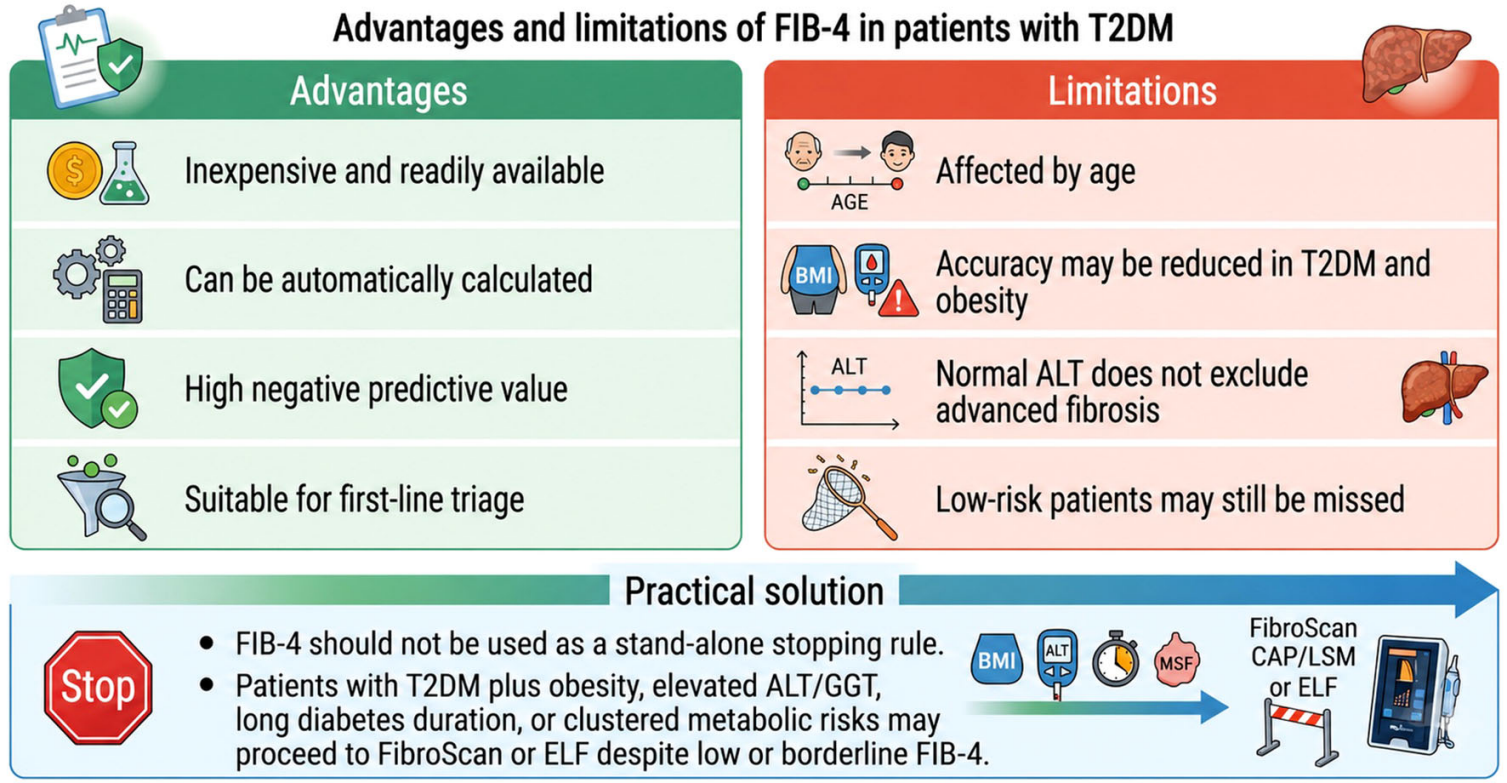
Advantages and limitations of FIB-4 in patients with T2DM. FIB-4 is inexpensive, readily available, suitable for automated calculation and useful for first-line triage. However, its performance may be affected by age, obesity, diabetes-related metabolic heterogeneity and aminotransferase variability. In patients with additional risk features, low or borderline FIB-4 should not be used as a stand-alone stopping rule, and FibroScan CAP/LSM or ELF may still be warranted.

Although FIB-4 is the most practical first-line tool for endocrinology outpatient screening, the result should not be interpreted mechanically. Age, obesity, T2DM-related metabolic heterogeneity and aminotransferase variability may affect performance. A low or borderline FIB-4 value does not fully exclude clinically significant fibrosis in selected patients with long-standing diabetes, central obesity, elevated ALT or GGT, thrombocytopenia, imaging-detected steatosis or clustered cardiometabolic risk factors. The practical advantages and limitations of FIB-4 are summarized in **Figure 4**.

The possibility of false-negative FIB-4 results deserves explicit attention. Guideline endorsement of FIB-4 reflects its accessibility and population-level usefulness, but it does not mean that FIB-4 has optimal diagnostic performance in every high-risk metabolic subgroup. Some patients with MRE-confirmed advanced fibrosis may still be classified as low risk by FIB-4-based pathways. In a recent cost-effectiveness analysis of high-risk metabolic liver disease populations, an ELF-based strategy using a threshold of 9.0 identified all individuals with MRE-confirmed advanced fibrosis in the evaluated cohort (40). These data support a more balanced approach: FIB-4 is a practical entry test, but patients with long-standing T2DM, obesity, abnormal liver biochemistry, thrombocytopenia, imaging-detected steatosis or clustered cardiometabolic risks may warrant ELF or elastography even when FIB-4 is low or borderline.

**Figure 4** underscores the main advantage and the main risk of FIB-4. Its strengths are accessibility, low cost, compatibility with electronic medical record automation and high negative predictive value in broad populations. These features make it well suited for first-line triage. At the same time, FIB-4 should not be used as a stand-alone stopping rule. In patients with T2DM and additional risk features, second-line assessment with FibroScan CAP/LSM or ELF may still be appropriate despite low or borderline FIB-4 values. This interpretation keeps the pathway feasible while reducing the likelihood of missing relevant fibrosis.

Patients with low-risk FIB-4 results should not be ignored. They should receive education regarding MASLD risk, lifestyle intervention, and optimization of cardiometabolic factors, including body weight, glycaemic control, blood pressure, triglycerides, and atherogenic dyslipidaemia. In these patients, repeat FIB-4 assessment at regular intervals is appropriate, particularly because T2DM is a dynamic disease and fibrosis risk may increase with ageing, weight gain, worsening insulin resistance, or progression of metabolic comorbidities. Current practice guidance generally supports periodic reassessment in patients with T2DM or multiple metabolic risk factors, even when baseline FIB-4 is low (13, 25). In an outpatient pathway, a 12-month reassessment interval may be practical for T2DM patients, with shorter reassessment considered when liver enzymes rise, platelet count declines, metabolic control worsens, or hepatic steatosis is documented on imaging.

Patients with indeterminate FIB-4 values should proceed to second-line non-invasive testing. This is the group in which a structured pathway provides the greatest value, because clinical uncertainty is common and inappropriate management can lead either to unnecessary referral or missed advanced fibrosis. Vibration-controlled transient elastography is the most practical second-line tool in many clinical settings because it provides liver stiffness measurement for fibrosis risk and controlled attenuation parameter for hepatic steatosis assessment. In a prospective analysis of patients with NAFLD, FibroScan-based CAP and liver stiffness measurement showed acceptable diagnostic performance for assessing steatosis and fibrosis, supporting its use as a non-invasive outpatient assessment tool (41). For endocrinology clinics, the practical advantage of FibroScan is that it can be completed quickly, does not require contrast or radiation, and produces results that can be linked directly to risk stratification and referral decisions.

Where FibroScan is not available within endocrinology clinics, ELF may be a more scalable second-line option because it can be ordered as a laboratory test, performed without specialized equipment at the point of care, and incorporated into existing laboratory workflows. Recent data comparing FIB-4 and ELF testing strategies in a veteran population suggest that ELF-based approaches can alter referral rates and costs, highlighting the need to choose the second-line test according to the clinical setting rather than assuming that elastography is always the easiest downstream assessment (42). For clinics without direct elastography access, an automated FIB-4 result followed by reflex or ordered ELF testing may provide a feasible bridge before hepatology referral.

The usefulness of ELF may be particularly relevant when alcohol exposure coexists with metabolic risk. In a primary-care alcohol pathway, ELF facilitated stratification of people with alcohol use disorder and helped identify those requiring specialist liver assessment (43). Although AUD populations are not identical to T2DM/MASLD cohorts, these data support the concept that a scalable blood-based fibrosis test can be useful when alcohol-related and metabolic liver risks overlap or when elastography access is limited.

The enhanced liver fibrosis test may be used as an alternative second-line test when elastography is unavailable, delayed, or technically unreliable. ELF is a serum-based fibrosis marker that reflects extracellular matrix turnover and has shown performance in identifying patients with NAFLD at increased risk of advanced fibrosis (44). Its use may be particularly useful in settings where FibroScan is not accessible to endocrinology clinics or where body habitus limits the reliability of elastography. However, ELF should also be interpreted within the broader clinical context, because no single non-invasive test can fully resolve diagnostic uncertainty. In a pragmatic pathway, FibroScan and ELF should be viewed as complementary tools rather than competing options.

In patients undergoing FibroScan, both CAP and LSM should be interpreted. CAP provides an estimate of hepatic steatosis, whereas LSM is more relevant for fibrosis risk. This distinction is important because the clinical objective of the pathway is not merely to confirm fat accumulation but to identify patients with clinically significant fibrosis. In selected cases, composite scores such as the FibroScan-AST score may provide additional information by combining liver stiffness, AST, and metabolic variables to identify patients at risk for active steatohepatitis with significant fibrosis (45). Such tools may be useful when the clinical question is whether the patient may have “at-risk MASH, “ but they should not complicate the pathway to the point that implementation becomes unrealistic. For routine endocrinology practice, FIB-4 followed by FibroScan or ELF remains the most feasible foundation.

Several non-invasive tools can be used within an endocrinology-based MASLD/MASH pathway, but they should not be treated as interchangeable. Each tool answers a different clinical question. FIB-4 is a low-cost first-line triage test; FibroScan CAP and LSM provide practical second-line assessment of steatosis and fibrosis risk; ELF may be useful when elastography is unavailable or technically unreliable; and MRI-PDFF or MRE should be reserved for selected high-risk, discordant or research-oriented cases. Their practical outpatient roles are summarized in **Table 3**.

**Table 3:** Comparison of non-invasive tools available for MASLD/MASH assessment in endocrinology outpatient clinics.

Tool	Required data	Main purpose	Advantages	Limitations	Practical use in endocrinology outpatient clinics
FIB-4	Age, AST, ALT, platelet count	First-line fibrosis risk stratification	Inexpensive; readily available; can be automatically calculated from routine laboratory data; high negative predictive value for excluding advanced fibrosis	Affected by age; reduced accuracy in some patients with T2DM or obesity; low-risk results may miss selected patients with clinically significant fibrosis	Best suited as the initial screening and triage tool. Should be embedded into the electronic medical record and used to determine whether second-line testing is needed (13, 24, 25)
NAFLD Fibrosis Score (NFS)	Age, BMI, impaired fasting glucose or diabetes, AST, ALT, platelet count, albumin	Blood-based estimation of advanced fibrosis risk	Uses routinely available clinical and biochemical variables; historically well studied in NAFLD	Includes BMI and diabetes variables, which may reduce discriminatory value in T2DM populations; relatively large indeterminate zone; less convenient than FIB-4	May be used as a supplementary fibrosis score, but FIB-4 is generally more practical for first-line outpatient screening (13)
APRI	AST, platelet count	Simple blood-based fibrosis assessment	Very simple; low cost; based on routine laboratory data	Lower diagnostic performance for MASLD-related advanced fibrosis than FIB-4 or sequential algorithms; not specific for MASLD/MASH	Not preferred as a primary tool in endocrinology clinics, but may provide supportive information when other scores are unavailable (13)
ELF test	Serum markers of extracellular matrix turnover, including hyaluronic acid, PIIINP, and TIMP-1	Second-line fibrosis risk assessment	Blood-based; useful when FibroScan is unavailable or technically limited; provides fibrosis-related risk information	Cost and availability vary; not always accessible in routine endocrine clinics; thresholds may differ across populations and settings	Suitable as a second-line test for patients with indeterminate or high FIB-4, especially where VCTE/FibroScan is unavailable (24, 44)
FibroScan CAP	Vibration-controlled transient elastography with controlled attenuation parameter	Non-invasive assessment of hepatic steatosis	Rapid; non-invasive; performed in outpatient settings; useful for identifying and monitoring hepatic steatosis	Less accurate in severe obesity or poor-quality examinations; cut-offs vary by population and probe type; does not directly stage inflammation	Useful as part of second-line assessment after FIB-4, especially for documenting steatosis burden and monitoring liver fat trend (41)
FibroScan LSM	Vibration-controlled transient elastography with liver stiffness measurement	Non-invasive assessment of fibrosis risk	Rapid; non-invasive; practical for outpatient risk stratification; helps identify patients requiring hepatology referral	Liver stiffness can be influenced by inflammation, congestion, cholestasis, food intake, and technical factors; unreliable measurements may occur in obesity	Core second-line test for patients with indeterminate or high FIB-4. Elevated LSM should prompt hepatology referral or further evaluation (13, 25, 41)
FAST score	FibroScan LSM, CAP, and AST	Identification of patients at risk for active steatohepatitis with significant fibrosis	Combines imaging and biochemical information; designed to enrich for “at-risk NASH/MASH” and reduce unnecessary biopsy	Requires FibroScan and AST; not a general screening tool for all T2DM patients; performance and thresholds require local interpretation	Useful in selected patients after FibroScan when the clinical question is whether active MASH with significant fibrosis is likely (45)
MRI-PDFF	Quantitative MRI-based proton density fat fraction	Quantitative measurement of liver fat content and treatment response	Highly quantitative; sensitive to change in liver fat; useful for clinical trials and research-oriented follow-up; good repeatability for monitoring MASLD/MASH	Expensive; less accessible; not suitable as a routine first-line screening test; does not directly stage fibrosis	Best suited for high-risk or research settings, or when quantitative monitoring of liver fat change is needed after lifestyle or pharmacological intervention (46, 47)
MRE	Magnetic resonance elastography	Quantitative assessment of liver stiffness and fibrosis risk	High diagnostic accuracy for advanced fibrosis; useful when VCTE is unreliable or results are discordant; can be combined with MRI-PDFF	Expensive; limited availability; requires MRI infrastructure and expertise	Appropriate for high-risk, diagnostically uncertain, or discordant cases, particularly when FibroScan is unreliable or advanced fibrosis is suspected (13, 34)
Conventional ultrasound	Abdominal ultrasonography	Qualitative detection of hepatic steatosis and exclusion of structural liver disease	Widely available; inexpensive; familiar to clinicians; can identify cirrhotic morphology, focal lesions, or biliary abnormalities	Limited sensitivity for mild steatosis; operator-dependent; cannot reliably quantify liver fat or stage fibrosis; normal ultrasound does not exclude MASLD/MASH	Useful for initial imaging or exclusion of structural disease, but should not replace fibrosis risk stratification with FIB-4 and second-line non-invasive tests (13)

ALT, alanine aminotransferase; APRI, AST-to-platelet ratio index; AST, aspartate aminotransferase; BMI, body mass index; CAP, controlled attenuation parameter; ELF, enhanced liver fibrosis test; FAST, FibroScan-AST score; FIB-4, fibrosis-4 index; LSM, liver stiffness measurement; MASLD, metabolic dysfunction-associated steatotic liver disease; MASH, metabolic dysfunction-associated steatohepatitis; MRE, magnetic resonance elastography; MRI-PDFF, magnetic resonance imaging-proton density fat fraction; NFS, NAFLD fibrosis score; PIIINP, procollagen III amino-terminal peptide; T2DM, type 2 diabetes mellitus; TIMP-1, tissue inhibitor of metalloproteinase-1; VCTE, vibration-controlled transient elastography.

As shown in **Table 3**, the main value of FIB-4 is accessibility and suitability for automated first-line triage, not definitive fibrosis staging. FibroScan LSM is more directly relevant to fibrosis risk and referral decisions, whereas CAP provides supportive information on steatosis. ELF can serve as a second-line alternative where elastography is not available. MRI-PDFF and MRE provide more quantitative assessment, but cost and availability limit their role in routine screening. A staged strategy—FIB-4 for broad triage, FibroScan or ELF for risk refinement, and MRI-PDFF or MRE for selected high-risk or uncertain cases—is therefore more practical than reliance on a single test.

A further distinction is needed between referral triage and fibrosis staging. In endocrinology or primary care, the immediate question is often whether a patient should be referred to hepatology; this use case favours tests that are sensitive, scalable and easy to order, such as FIB-4 followed by ELF in settings where elastography is not readily available. After referral, the hepatology question is different: the specialist must stage fibrosis severity, assess cirrhosis risk, determine surveillance needs and evaluate treatment eligibility. This second use case may require FibroScan LSM, MRE and, in selected cases, liver biopsy. Thus, ELF and VCTE should not be described as fully interchangeable; they may occupy different positions depending on whether the goal is referral triage or specialist staging.

High-risk patients require a different level of evaluation. This group includes patients with FIB-4 above the high-risk threshold, markedly elevated LSM, discordant or concerning non-invasive test results, thrombocytopenia, imaging signs suggestive of cirrhosis or portal hypertension, or persistent unexplained liver enzyme elevation. These patients should be referred to hepatology or evaluated in a multidisciplinary metabolic liver clinic where available. The purpose of referral is not only to confirm fibrosis stage, but also to assess for cirrhosis, portal hypertension, hepatocellular carcinoma risk, competing liver diseases, and eligibility for liver-directed therapy. In selected high-risk or diagnostically uncertain patients, MRI-PDFF and magnetic resonance elastography may provide more quantitative assessment of hepatic fat and fibrosis. MRI-PDFF has also been used as a non-invasive biomarker of treatment response, and a relative reduction of at least 30% has been associated with histological response in NASH studies (46). Nevertheless, MRI-based testing is costly and should be reserved for patients in whom the result is likely to change management, or for research-oriented follow-up.

Once risk categories have been assigned, management should be matched to fibrosis risk rather than delivered as generic advice. For low-risk patients, management should focus on lifestyle intervention, weight stabilization or weight loss where appropriate, cardiometabolic risk reduction, and periodic reassessment. For intermediate-risk patients, the same metabolic interventions are required, but follow-up should be more deliberate and should include repeat FIB-4 and, where appropriate, repeat elastography. For high-risk patients, hepatology co-management is needed, and liver-directed evaluation should proceed in parallel with intensified metabolic treatment. This risk-stratified model avoids two common errors: reassuring all patients with “fatty liver” as if the condition were benign, and referring all patients with steatosis regardless of fibrosis risk.

Lifestyle intervention remains the foundation of management across all risk categories. Histological studies have shown that greater weight loss is associated with greater improvement in NASH features, and weight loss of approximately 10% or more is associated with the highest rates of NASH resolution and fibrosis regression (48). In the endocrinology clinic, however, lifestyle advice should be concrete and measurable. It should be linked to body weight, waist circumference, HbA1c, triglycerides, blood pressure, and liver-related indicators rather than expressed as unspecific recommendations to “eat less and exercise more.” Patients should understand that the aim is not only glucose control, but reduction of hepatic and systemic metabolic risk.

Pharmacological treatment should also be aligned with the patient’s metabolic and liver risk profile. For patients with T2DM and MASLD, sodium-glucose cotransporter 2 inhibitors and glucagon-like peptide-1 receptor agonists may be particularly attractive when there are additional indications such as obesity, cardiovascular disease, heart failure, or chronic kidney disease. In the E-LIFT trial, empagliflozin reduced liver fat measured by MRI-PDFF in patients with T2DM and NAFLD (49). Semaglutide has shown histological benefit in MASH trials, and a phase 3 trial reported significant improvement in steatohepatitis resolution and fibrosis-related endpoints in patients with MASH and fibrosis (50). Tirzepatide has also demonstrated efficacy for MASH resolution without worsening fibrosis in patients with moderate or severe fibrosis (51). These data support the concept that metabolic pharmacotherapy can be incorporated into a MASLD/MASH pathway, although medication choices should still be individualized according to regulatory approval, comorbidities, tolerability, cost, and specialist input.

Disease-specific liver therapy is becoming increasingly relevant for high-risk MASH, but it should not be introduced into endocrine practice without appropriate staging and specialist collaboration. Resmetirom, a thyroid hormone receptor-β selective agonist, showed benefit in a phase 3 trial of patients with NASH and liver fibrosis (52). Such developments make early identification of at-risk patients more clinically meaningful, because patients with significant fibrosis may now have potential access to disease-directed therapy in addition to lifestyle and metabolic treatment. However, these agents require careful patient selection and monitoring; therefore, their role in an endocrinology-based pathway should be to strengthen referral and co-management rather than replace hepatology involvement.

The final step is longitudinal follow-up. A pathway without follow-up is merely a screening exercise. For patients with low-risk FIB-4, annual or periodic reassessment using routine laboratory data may be sufficient, together with reinforcement of lifestyle and metabolic goals. For intermediate-risk patients, 24-week follow-up can assess early metabolic response, liver enzyme trends, body weight, waist circumference, HbA1c, triglycerides, and treatment adherence; 48-week follow-up can evaluate more durable changes and determine whether repeat FibroScan or ELF testing is needed. For high-risk patients, follow-up should be coordinated with hepatology and may include repeat elastography, MRI-PDFF or MRE where appropriate, cirrhosis surveillance if indicated, and reassessment of liver-directed treatment eligibility. The 24-week and 48-week time points are clinically practical because they correspond to common intervals used in metabolic intervention studies and allow enough time to observe meaningful changes in weight, glycaemic control, liver fat, and aminotransferase levels.

The proposed 24-week and 48-week time points should be interpreted as pragmatic follow-up checkpoints rather than mandatory guideline-derived intervals. A 24-week visit is intended to capture early adherence, weight change, glycaemic control, medication optimization and completion of second-line testing or referral. A 48-week visit provides a practical annual reassessment point for repeating routine metabolic variables and, when available, liver-related indicators such as CAP, LSM, FIB-4 or MRI-PDFF. These intervals were selected to align liver-risk reassessment with routine diabetes follow-up and to provide measurable quality indicators for implementation studies; they should be shortened or extended according to baseline risk, local resources and clinical judgement.

In summary, an endocrinology outpatient pathway for MASLD/MASH in T2DM should begin with automated FIB-4 calculation, proceed to FibroScan CAP/LSM or ELF testing in patients who need risk refinement, direct high-risk patients to hepatology evaluation, and return all patients to structured metabolic management and follow-up. The pathway is not a substitute for hepatology assessment; it is a front-line triage and management framework for the diabetes clinic.

## Validation indicators for the proposed pathway

A risk-stratification pathway is useful only if it can be evaluated. For MASLD/MASH screening in endocrinology outpatient clinics, validation should not be limited to diagnostic accuracy. A clinically valuable pathway must also show that eligible patients are screened, second-line testing is completed, high-risk patients are referred, interventions are implemented, and follow-up is sustained.

Because the proposed pathway is intended for routine outpatient use, validation should include implementation and clinical dimensions. Key domains include feasibility, screening yield, metabolic and liver-related response, management quality, follow-up retention and long-term outcomes. A concise set of validation indicators is provided in **Table 4**.

**Table 4:** Validation indicators for an endocrinology outpatient MASLD/MASH risk-stratification pathway.

Indicator category	Core indicators	Calculation/definition	Assessment time point	Significance
Feasibility	FIB-4 calculation rate; complete-data availability; FibroScan/ELF completion rate; MRI-PDFF/MRE completion rate; hepatology referral completion rate	Completed or available assessments divided by eligible or indicated patients	Baseline; monthly or quarterly quality review; within 4-12 weeks for second-line testing or referral	Measures whether the pathway can be implemented in routine outpatient care
Screening performance	FIB-4 risk-category distribution; high-risk case detection rate; LSM >=8, >=10 or >=12 kPa; MRI-PDFF >=5%; discordant NIT rate	Number or proportion meeting each predefined risk or test threshold	Baseline and after second-line testing	Describes disease burden, referral load and the yield of sequential risk stratification
Metabolic improvement	Body weight, waist circumference, HbA1c, TG, HDL-C, LDL-C, ALT, AST and GGT	Absolute and percentage change from baseline	24 weeks; 48 weeks; annual reassessment	Assesses response to lifestyle intervention, medication optimization and cardiometabolic risk reduction
Liver-related improvement	CAP, LSM, MRI-PDFF, FIB-4 value and risk-category change	Absolute change, relative change, or change in risk category	24 weeks for liver-fat response when available; 48 weeks or longer for fibrosis-risk trajectory	Tracks hepatic steatosis and fibrosis-related risk over time
Management quality	Lifestyle counselling documentation; lifestyle implementation rate; medication optimization rate; patient adherence	Documented or completed care processes divided by eligible patients	Baseline; 24 weeks; 48 weeks	Shows whether risk-adapted care is actually delivered after screening
Follow-up retention	24-week follow-up rate; 48-week follow-up rate; loss-to-follow-up rate	Completed follow-up visits divided by scheduled or enrolled patients	24 and 48 weeks	Measures pathway sustainability and patient engagement
Long-term outcomes	Progression to advanced fibrosis; cirrhosis; HCC; cardiovascular events; CKD progression; all-cause mortality	Incident clinical outcomes confirmed by clinical, imaging, laboratory or specialist assessment	Annual and long-term follow-up	Links outpatient screening and management to clinically meaningful endpoints

ALT, alanine aminotransferase; AST, aspartate aminotransferase; CAP, controlled attenuation parameter; CKD, chronic kidney disease; eGFR, estimated glomerular filtration rate; ELF, enhanced liver fibrosis test; FIB-4, fibrosis-4 index; GGT, gamma-glutamyl transferase; HbA1c, glycated haemoglobin; HCC, hepatocellular carcinoma; HDL-C, high-density lipoprotein cholesterol; LDL-C, low-density lipoprotein cholesterol; LSM, liver stiffness measurement; MASLD, metabolic dysfunction-associated steatotic liver disease; MASH, metabolic dysfunction-associated steatohepatitis; MRE, magnetic resonance elastography; MRI-PDFF, magnetic resonance imaging-proton density fat fraction; T2DM, type 2 diabetes mellitus; TG, triglycerides.

**Table 4** frames the pathway as a staged and multidimensional clinical system. Feasibility indicators determine whether the pathway can be implemented in daily practice. Screening indicators describe risk distribution and the yield of second-line testing. Clinical response indicators assess whether metabolic and liver-related measures improve after intervention. Follow-up and management indicators determine whether the pathway is sustained. Long-term outcomes, including advanced fibrosis, cirrhosis, hepatocellular carcinoma, cardiovascular events, CKD progression and mortality, provide the basis for future prospective validation.

Feasibility and screening performance should be assessed first. Core metrics include complete-data availability, automated FIB-4 calculation rate, FibroScan or ELF completion rate, hepatology referral completion rate and the distribution of low-, indeterminate- and high-risk categories. These indicators show whether the pathway can operate in routine care and what clinical yield it produces.

Clinical response should be evaluated through both metabolic and liver-related measures. Body weight, waist circumference, HbA1c, lipid profile and liver enzymes provide accessible metabolic and biochemical endpoints, while CAP, LSM, FIB-4 and MRI-PDFF, when available, help describe changes in hepatic steatosis and fibrosis-related risk. A 24-week assessment is appropriate for early metabolic and liver-fat response, whereas 48-week and annual reassessment are more informative for durability and fibrosis-risk trajectory.

Management quality should capture whether care is delivered consistently, not merely whether risk was detected. Relevant indicators include lifestyle counselling documentation, lifestyle implementation rate, medication optimization rate, patient adherence, 24-week and 48-week follow-up completion and loss to follow-up. These measures are particularly important because a screening pathway has little value if second-line testing, referral or follow-up is not completed.

Accordingly, the 24-week and 48-week indicators in **Table 4** are proposed process measures for implementation evaluation, not evidence-based thresholds for liver outcome improvement. They are intended to help clinics determine whether screening led to action, referral and reassessment.

Long-term outcomes should include progression to advanced fibrosis, cirrhosis, hepatocellular carcinoma, cardiovascular events, CKD progression and mortality. Although many outpatient studies will not be powered for these outcomes initially, specifying them allows future cohorts and quality-improvement projects to connect short-term process indicators with clinically meaningful endpoints.

Overall, validation should remain multidimensional. Feasibility indicators show whether the pathway can be implemented; screening indicators show whether it identifies relevant risk; clinical indicators show whether management changes patient trajectories; follow-up indicators show whether the pathway is sustainable; and long-term outcomes determine whether earlier risk identification translates into better care.

## Treatment and follow-up strategies within the pathway

Treatment is included in this pathway only insofar as it affects risk communication, referral decisions and follow-up planning. The purpose of this review is not to provide a comprehensive pharmacotherapy review, but to show how liver-risk stratification can guide the intensity and coordination of metabolic care.

Lifestyle intervention and sustained weight reduction remain the foundation of management for patients with T2DM and MASLD/MASH. A weight loss target of 7–10% is commonly associated with improvement in steatohepatitis-related activity, while larger and sustained reductions are more likely to affect fibrosis risk. In endocrinology clinics, this means that a low-risk liver profile should still lead to structured weight, glycaemic, blood pressure and lipid management rather than simple reassurance.

Among glucose-lowering agents, SGLT2 inhibitors and GLP-1 receptor agonists are most relevant to the pathway because they address glycaemia, body weight and cardiometabolic risk, with evidence of improvement in hepatic steatosis or MASH-related activity in selected studies. Tirzepatide has also shown histological benefit in patients with MASH and moderate or severe fibrosis. These agents should be selected according to the patient’s metabolic phenotype and comorbidities, not solely because steatosis is present.

Pioglitazone may remain useful in selected patients with T2DM and biopsy-proven or strongly suspected MASH, but its risks, including weight gain, oedema, fracture risk and heart failure susceptibility, require individualized assessment. Statins should not be withheld when indicated for cardiovascular risk reduction. Resmetirom and other liver-directed therapies make accurate identification of at-risk MASH more clinically consequential, but their use requires appropriate staging and hepatology collaboration.

Accordingly, pharmacological decisions within the pathway should be risk-adapted: low-risk patients require metabolic prevention and reassessment; intermediate-risk patients require intensified metabolic treatment and second-line liver assessment; and high-risk patients require hepatology co-management in parallel with optimization of diabetes, weight and cardiometabolic risk. The main intervention options and liver-related evidence are summarized in **Table 5**.

**Table 5:** Intervention strategies and liver-related evidence for patients with T2DM and MASLD/MASH.

Intervention	Suitable population	Main effects	Liver-related evidence	Practical recommendation in endocrinology outpatient clinics
Lifestyle intervention	All patients with T2DM and MASLD/MASH, regardless of fibrosis risk category	Improves insulin resistance, body weight, visceral adiposity, glycaemic control, lipid profile, blood pressure, and systemic metabolic risk	Lifestyle-induced weight loss is associated with improvement in NASH activity; greater weight loss, especially around 10% or more, is associated with higher rates of NASH resolution and fibrosis regression (48)	Should be the foundation of management. Advice should be structured, measurable, and linked to weight, waist circumference, HbA1c, liver enzymes, and follow-up indicators rather than given as nonspecific lifestyle counselling
Weight reduction/obesity management	Patients with overweight, obesity, central adiposity, or obesity-related metabolic risk	Reduces hepatic fat accumulation, improves insulin resistance, lowers inflammatory and cardiometabolic burden	Weight reduction improves hepatic steatosis and MASH-related histological features; more substantial and sustained weight loss is associated with greater liver benefit (48)	Set realistic weight-loss targets, monitor body weight and waist circumference at 24 and 48 weeks, and consider anti-obesity pharmacotherapy or multidisciplinary obesity care when lifestyle intervention alone is insufficient
SGLT2 inhibitors	Patients with T2DM and MASLD/MASH, especially those with obesity, heart failure risk, CKD, or high cardiometabolic risk	Improves glycaemic control; modestly reduces body weight; provides cardiorenal benefit in appropriate patients; may reduce liver fat	Empagliflozin reduced liver fat measured by MRI-PDFF in patients with T2DM and NAFLD/MASLD, while changes in fibrosis scores such as FIB-4 or NFS may be less evident over short follow-up. Meta-analytic evidence supports improvement in hepatic steatosis-related outcomes, although fibrosis effects remain less certain (49, 53, 54)	Consider when clinically indicated for diabetes, CKD, heart failure, or cardiometabolic risk. MRI-PDFF or CAP may be more sensitive than FIB-4/NFS for detecting short-term liver fat response
GLP-1 receptor agonists	Patients with T2DM and MASLD/MASH, particularly those with obesity, need for weight reduction, or high cardiovascular risk	Promotes weight loss, improves glycaemic control, reduces appetite and cardiometabolic risk; may improve MASH activity	Semaglutide has shown histological benefit in MASH. In a phase 3 trial, once-weekly semaglutide 2.4 mg improved steatohepatitis resolution and fibrosis-related endpoints in patients with MASH and F2/F3 fibrosis (50)	Useful when weight reduction and glycaemic control are major clinical priorities. In patients with intermediate or high liver risk, combine use with structured liver follow-up rather than relying only on HbA1c or body weight
Dual GIP/GLP-1 receptor agonist, tirzepatide	Patients with T2DM or obesity and MASLD/MASH; particularly relevant when weight loss and metabolic improvement are priorities	Produces substantial weight loss, improves glycaemic control and insulin resistance, and may reduce MASH activity	In a 52-week phase 2 trial in biopsy-confirmed MASH with F2/F3 fibrosis, tirzepatide produced higher rates of MASH resolution without worsening of fibrosis than placebo; fibrosis improvement was also observed as a key secondary endpoint (51)	Promising for patients whose MASLD/MASH is driven by obesity and insulin resistance. High-risk fibrosis or suspected cirrhosis still requires hepatology co-management
Pioglitazone	Selected patients with T2DM or prediabetes and biopsy-proven or strongly suspected MASH, particularly when other options are unsuitable	Improves insulin sensitivity and may improve steatohepatitis; may increase weight and fluid retention	Long-term pioglitazone treatment improved NASH histological outcomes in patients with prediabetes or T2DM and NASH (55)	Can be considered in selected patients after individualized risk-benefit assessment. Use cautiously in patients at risk of weight gain, oedema, heart failure, or fracture
Statins	Patients with T2DM and MASLD/MASH who have dyslipidaemia or elevated ASCVD risk	Reduces LDL-C and ASCVD risk; may improve liver enzymes in some patients; not primarily an anti-MASH or anti-fibrotic therapy	Statins are generally safe in NAFLD/MASLD, including in patients with mildly elevated liver enzymes, and cardiovascular benefits usually outweigh concerns about liver toxicity (56)	Do not withhold statins solely because of MASLD or mildly abnormal aminotransferases. Use according to cardiovascular risk and lipid guidelines, while continuing liver risk stratification separately
Resmetirom	Adults with noncirrhotic MASH/NASH and moderate-to-advanced fibrosis, generally requiring specialist assessment	Liver-directed thyroid hormone receptor-β agonist; reduces liver fat and improves MASH and fibrosis-related histological endpoints	In a phase 3 trial, resmetirom improved NASH resolution and fibrosis endpoints compared with placebo (52). The FDA approved resmetirom in 2024 for adults with noncirrhotic NASH with moderate-to-advanced fibrosis, in conjunction with diet and exercise (57)	Should be managed through hepatology or multidisciplinary metabolic liver clinics. Endocrinology clinics should identify and refer potentially eligible high-risk patients rather than initiate liver-directed therapy independently
Hepatology referral/multidisciplinary care	Patients with high-risk FIB-4, elevated LSM or ELF, suspected advanced fibrosis/cirrhosis, discordant non-invasive tests, unexplained liver enzyme abnormalities, or possible competing liver disease	Confirms fibrosis stage; excludes alternative liver diseases; evaluates cirrhosis, portal hypertension, HCC risk, and eligibility for liver-directed therapy	Current guidelines and pathways recommend referral or specialist evaluation for high-risk non-invasive test results or suspected advanced fibrosis (13, 24, 25)	Establish predefined referral criteria and feedback loops. Referral should not interrupt endocrine management; metabolic therapy, cardiovascular risk reduction, and liver staging should proceed in parallel

ASCVD, atherosclerotic cardiovascular disease; CAP, controlled attenuation parameter; CKD, chronic kidney disease; ELF, enhanced liver fibrosis test; FIB-4, fibrosis-4 index; GIP, glucose-dependent insulinotropic polypeptide; GLP-1, glucagon-like peptide-1; HbA1c, glycated haemoglobin; HCC, hepatocellular carcinoma; LDL-C, low-density lipoprotein cholesterol; LSM, liver stiffness measurement; MASLD, metabolic dysfunction-associated steatotic liver disease; MASH, metabolic dysfunction-associated steatohepatitis; MRE, magnetic resonance elastography; MRI-PDFF, magnetic resonance imaging-proton density fat fraction; NAFLD, non-alcoholic fatty liver disease; NASH, non-alcoholic steatohepatitis; NFS, NAFLD fibrosis score; T2DM, type 2 diabetes mellitus.

The proposed pathway should not end with risk classification. For patients with T2DM and MASLD/MASH, screening has clinical value only when it leads to risk-adapted intervention and follow-up. Endocrinology clinics are central to this step because they already manage weight, glycaemic control, dyslipidaemia, blood pressure and cardiorenal risk. The challenge is to link these metabolic decisions to liver-related endpoints rather than treating MASLD/MASH as a disconnected comorbidity.

Lifestyle intervention remains the foundation of MASLD/MASH management. This recommendation is often repeated so frequently that it can sound formulaic, but the evidence is clinically important. In biopsy-confirmed NASH, weight loss through lifestyle modification was associated with dose-dependent improvements in steatohepatitis and fibrosis, with the greatest histological benefit observed in patients who achieved substantial weight reduction (48). For patients with T2DM, lifestyle intervention also improves glycaemic control, visceral adiposity, blood pressure, triglycerides and overall cardiometabolic risk.

Dietary counselling, physical activity, and weight reduction should be adapted to the patient’s age, physical capacity, diabetes duration, cardiovascular risk, sarcopenia risk, and medication profile. In patients with obesity or central adiposity, intentional weight loss is usually a reasonable therapeutic goal. In older or frail patients, however, weight loss should not be pursued at the expense of muscle mass, nutrition, or functional status. Therefore, a clinic pathway should avoid a uniform lifestyle prescription. Instead, it should define achievable targets, document baseline weight and waist circumference, reassess them at 24 and 48 weeks, and use these data to decide whether additional pharmacological or multidisciplinary support is needed.

Pharmacological therapy in T2DM complicated by MASLD/MASH should be selected according to the patient’s overall metabolic and organ-risk profile. SGLT2 inhibitors have attracted attention because they improve glycaemic control, reduce body weight modestly, and have established cardiovascular and renal benefits in appropriate patients. In the E-LIFT randomized trial, empagliflozin reduced liver fat measured by MRI-PDFF in patients with T2DM and NAFLD (49). A more recent systematic review and meta-analysis further suggested that SGLT2 inhibitors improve hepatic steatosis-related outcomes in patients with NAFLD/MASLD, although the magnitude of benefit varies across studies and fibrosis endpoints remain less certain (53). Thus, SGLT2 inhibitors may be particularly suitable for patients with T2DM and MASLD who also have heart failure risk, chronic kidney disease, obesity, or high cardiometabolic burden. Their role in the pathway should be viewed primarily as metabolic and organ-protective therapy with potential hepatic benefits, rather than as stand-alone anti-fibrotic treatment.

GLP-1 receptor agonists occupy a similarly important position, especially in patients with obesity, poor weight control, or high cardiovascular risk. Semaglutide has shown histological benefit in MASH trials, and phase 3 evidence has further strengthened the rationale for incretin-based therapy in patients with MASH and fibrosis (50). In an endocrinology pathway, GLP-1 receptor agonists are attractive because they directly address weight, glycaemia, cardiometabolic risk, and liver fat-related disease activity. Nevertheless, their use should be individualized. Not every patient with steatosis requires a GLP-1 receptor agonist, and not every biochemical improvement should be interpreted as resolution of MASH or fibrosis. When GLP-1 receptor agonists are used in patients with intermediate or high liver risk, follow-up should include not only HbA1c and body weight, but also liver enzymes and, where available, CAP, LSM, or MRI-PDFF.

Dual GIP/GLP-1 receptor agonism has added a further therapeutic dimension. In a randomized phase 2 trial, tirzepatide significantly increased the proportion of patients with MASH resolution without worsening fibrosis among individuals with MASH and moderate or severe fibrosis (51). This finding is clinically relevant for endocrinologists because tirzepatide is already used in metabolic practice for T2DM and obesity in many settings. However, the interpretation should remain careful. Tirzepatide may be highly relevant for patients whose liver risk is driven by obesity, insulin resistance, and metabolic inflammation, but high-risk patients with suspected advanced fibrosis or cirrhosis still require hepatology evaluation. In other words, incretin-based therapy can be integrated into the pathway, but it should not replace fibrosis staging or specialist referral when referral criteria are met.

Pioglitazone remains relevant in selected patients with T2DM and biopsy-proven or strongly suspected MASH, although its use requires careful attention to weight gain, fluid retention, fracture risk, and heart failure susceptibility. A randomized trial showed that long-term pioglitazone treatment was effective in patients with prediabetes or T2DM and NASH (55). Because many endocrinologists are familiar with pioglitazone, it may still have a place in carefully selected patients, particularly when other options are unavailable or unsuitable. However, in contemporary practice, the decision to use pioglitazone should be individualized and should consider competing cardiometabolic risks, patient preference, and the availability of agents with weight-reducing and cardiorenal benefits.

For patients with high-risk MASH or significant fibrosis, hepatology collaboration is essential. The role of the endocrinologist is to identify risk, optimize metabolic treatment, and ensure referral, but not to manage advanced liver disease in isolation. Patients with high FIB-4, elevated LSM, suspected cirrhosis, thrombocytopenia, discordant non-invasive tests, or signs of portal hypertension should be evaluated by hepatology according to local referral pathways (13, 24, 25). Specialist assessment is needed to exclude competing liver diseases, confirm fibrosis severity, evaluate the need for hepatocellular carcinoma surveillance or variceal screening, and determine whether liver-directed therapy is appropriate. This is increasingly important because disease-specific pharmacotherapy is now entering clinical practice. Resmetirom improved both MASH resolution and fibrosis endpoints in a phase 3 trial, and the US FDA approved resmetirom in 2024 for adults with noncirrhotic NASH/MASH with moderate to advanced fibrosis, to be used together with diet and exercise (52, 57). These developments make accurate identification of patients with F2–F3 fibrosis more clinically consequential than before.

Treatment within an endocrinology-based MASLD/MASH pathway should be aligned with fibrosis risk, metabolic phenotype, comorbidities and the availability of hepatology collaboration. Lifestyle intervention and weight reduction remain the foundation of care, while SGLT2 inhibitors, GLP-1 receptor agonists and dual GIP/GLP-1 receptor agonists may provide additional metabolic and liver-related benefits in selected patients. Liver-directed therapies and management of advanced fibrosis require accurate staging and specialist input. The main intervention options and liver-related evidence are summarized in **Table 5**.

**Table 5** should therefore be read as a decision-support summary rather than as a separate drug review. Most interventions used in endocrinology clinics act by improving weight, insulin resistance, glycaemic control and cardiometabolic risk; liver-specific effects should be interpreted in relation to baseline fibrosis risk and the availability of follow-up measures such as CAP, LSM or MRI-PDFF.

Follow-up should be risk-adapted. Low-risk patients can usually undergo repeated FIB-4 assessment and routine metabolic follow-up. Intermediate-risk patients should be reassessed at approximately 24 weeks for body weight, waist circumference, HbA1c, triglycerides, ALT, AST, GGT, treatment adherence and, when obtained at baseline, CAP or MRI-PDFF. High-risk patients require verification that hepatology referral, further staging and liver-specific management have been completed. The 48-week time point is more suitable for evaluating durability of metabolic improvement and the need for repeat FibroScan, ELF, MRI-PDFF or hepatology reassessment.

In summary, treatment and follow-up within an endocrinology-based MASLD/MASH pathway should remain focused on the risk-stratification goal of the article. Lifestyle intervention is foundational; metabolic pharmacotherapy should be selected according to glycaemic, weight, cardiovascular, renal and liver-risk profiles; and high-risk patients should enter hepatology co-management. This integrates liver care into diabetes management without asking endocrinology clinics to replace hepatology services.

**Table 5** emphasizes an important distinction. Most interventions used in endocrinology clinics act mainly by improving body weight, insulin resistance, glycaemic control and cardiometabolic risk, rather than by directly reversing fibrosis. SGLT2 inhibitors may reduce liver fat over short to medium follow-up, but FIB-4 or NFS may not change substantially within 24 weeks, making MRI-PDFF or CAP more sensitive for early hepatic fat response. Incretin-based therapies, including GLP-1 receptor agonists and dual GIP/GLP-1 receptor agonists, have stronger evidence for weight loss and MASH-related histological improvement. Patients with suspected advanced fibrosis or cirrhosis still require hepatology evaluation. Resmetirom and other liver-directed therapies further increase the importance of identifying at-risk MASH, but their use should be coordinated with hepatology rather than handled as routine endocrine prescribing.

Follow-up should be risk-adapted rather than uniform. For low-risk patients, repeated FIB-4 assessment and routine metabolic follow-up may be sufficient, provided that reassessment is not forgotten. For intermediate-risk patients, 24-week follow-up should focus on early response to lifestyle and pharmacological intervention, including body weight, waist circumference, HbA1c, triglycerides, ALT, AST, GGT, and treatment adherence. If baseline CAP or MRI-PDFF was obtained, these measures can help quantify changes in hepatic steatosis. For high-risk patients, 24-week follow-up should additionally verify whether hepatology referral was completed and whether further staging or liver-specific management has been arranged. The 48-week time point is more suitable for assessing durability of metabolic improvement and determining whether repeat FibroScan, ELF, MRI-PDFF, or hepatology reassessment is needed.

Different non-invasive tools should have different roles during follow-up. MRI-PDFF is the most quantitative non-invasive measure of liver fat and has been associated with histological response in NASH studies, making it valuable for selected patients, clinical trials, and research-oriented pathways (46). However, because of cost and availability, MRI-PDFF should not be required for routine follow-up of all patients with T2DM and MASLD. CAP is more accessible when FibroScan is available and can provide a practical estimate of hepatic steatosis, although it should be interpreted with attention to technical quality and body habitus (41). LSM is more directly relevant to fibrosis risk and long-term liver-related outcomes. Longitudinal data suggest that changes in LSM are associated with liver-related event risk, supporting its use as a follow-up marker in patients with elevated baseline fibrosis risk (58). Thus, MRI-PDFF is best used to quantify liver fat response, CAP to provide accessible steatosis monitoring, and LSM to track fibrosis-related risk over time.

A conceptual shift is needed in clinical practice: the goal should not simply be to find fatty liver. In patients with T2DM, steatosis is common, and its detection alone rarely changes long-term management. The clinically important task is to identify significant fibrosis, advanced fibrosis and at-risk MASH, because these states alter prognosis, referral needs, surveillance strategies and therapeutic options.

In summary, treatment and follow-up within an endocrinology-based MASLD/MASH pathway should be structured, risk-adapted and outcome-oriented. Lifestyle intervention remains the foundation; metabolic pharmacotherapy should be selected according to glycaemic, weight, cardiovascular, renal and liver-risk profiles; and high-risk patients should enter hepatology co-management. Follow-up should combine accessible routine indicators with selected liver-specific measures such as CAP, LSM or MRI-PDFF when clinically useful. This approach integrates liver care into diabetes management without asking endocrinology clinics to replace hepatology services.

## Future directions

The proposed endocrinology outpatient pathway should be viewed as a starting framework rather than a fixed model. As MASLD/MASH screening moves from guideline statements into routine diabetes care, several issues require further work: electronic automation, multidisciplinary coordination, population-specific thresholds, prospective validation and integration into hospital-level quality improvement. These are not technical details; they will determine whether MASLD/MASH risk stratification becomes a sustainable part of chronic T2DM management.

**Figure 5:**
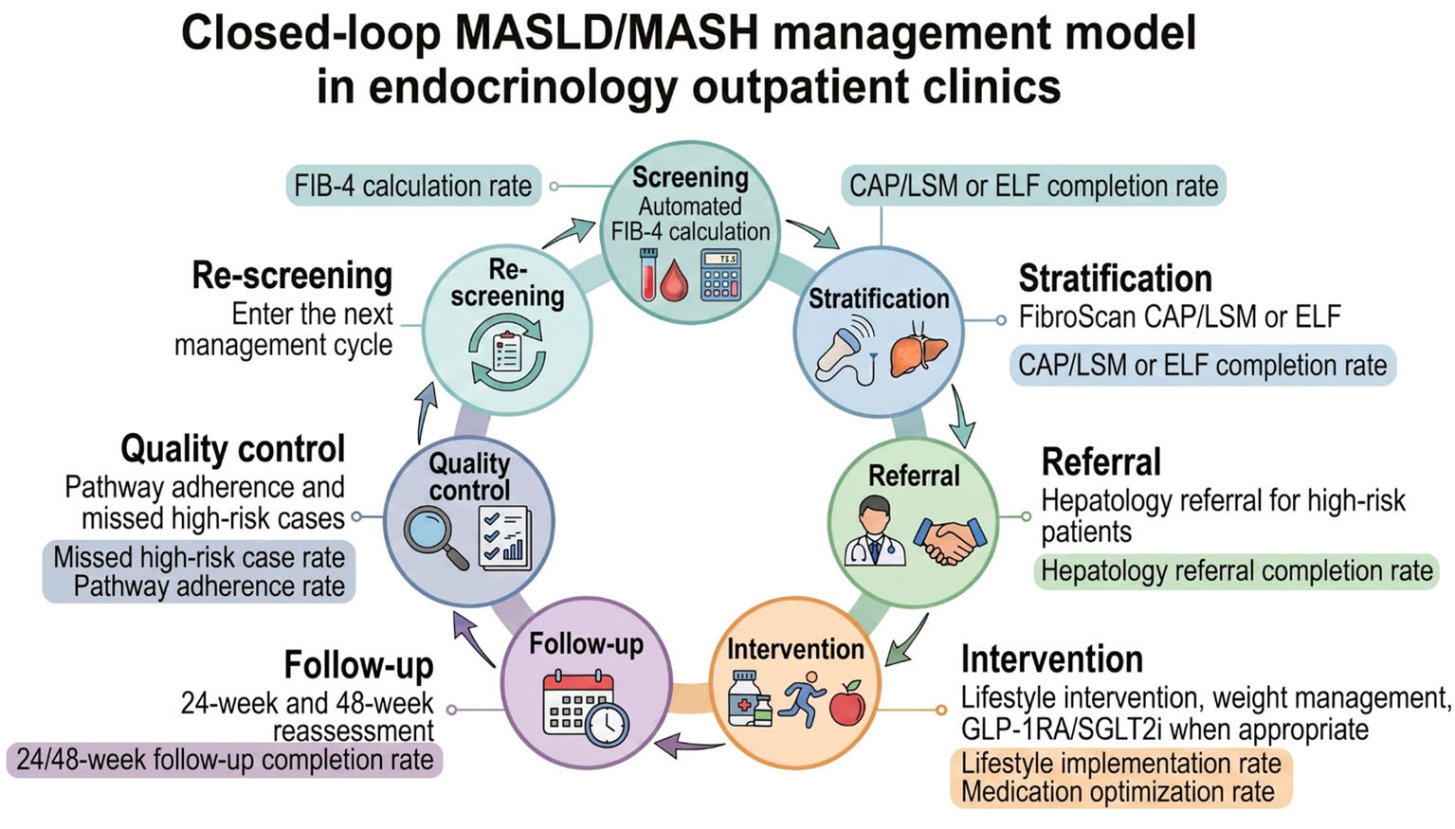
Closed-loop MASLD/MASH management model in endocrinology outpatient clinics. The model links automated FIB-4-based screening, second-line stratification with FibroScan CAP/LSM or ELF, hepatology referral for high-risk patients, lifestyle and pharmacological intervention, 24-week and 48-week reassessment, quality control and re-screening. Key process indicators include FIB-4 calculation rate, second-line testing completion rate, referral completion rate, lifestyle implementation rate, medication optimization rate, follow-up completion rate, missed high-risk case rate and pathway adherence rate.

For the pathway to become part of routine diabetes care, it should function as a closed-loop management system rather than a one-time screening algorithm. Automated FIB-4-based screening, second-line fibrosis stratification, hepatology referral, metabolic intervention, longitudinal reassessment, quality control and re-screening must be connected. This model is particularly relevant to real-world studies and hospital-level quality improvement, where success depends not only on detecting high-risk patients but also on completing downstream testing, referral, treatment alignment and follow-up. The proposed closed-loop model is illustrated in **Figure 5**.

**Figure 5** presents the pathway as a dynamic clinical system rather than a sequence of isolated tests. Screening begins with automated FIB-4 calculation, but the value of screening depends on completion of FibroScan CAP/LSM or ELF testing in patients with indeterminate or high-risk results. High-risk patients should enter a defined hepatology referral pathway, while metabolic intervention and pharmacological optimization continue in endocrinology care. Follow-up at 24 and 48 weeks provides an opportunity to reassess weight, glycaemic control, liver enzymes, CAP, LSM, MRI-PDFF when available, and referral adherence. Quality-control indicators can then inform re-screening and the next management cycle.

The first priority is electronic medical record-based FIB-4 automation and risk alerts. FIB-4 uses routine variables, but its clinical value is reduced if clinicians must calculate it manually during busy outpatient visits. Automated calculation can turn FIB-4 from a passive formula into an active screening tool. Ideally, once age, AST, ALT and platelet count are available for a patient with T2DM, the electronic system should calculate FIB-4, assign a risk category and provide a simple recommendation for follow-up, second-line testing or referral. Evidence from automated fibrosis score calculation and electronic reminder interventions suggests that such tools can improve recognition and management of patients with abnormal fibrosis scores (59). Electronic decision-support models may also support risk assessment when they are transparent, externally validated and calibrated to the local population (60, 61).

However, electronic automation should be designed carefully. An alert that simply reports an elevated FIB-4 without guidance may increase awareness but not necessarily improve care. Conversely, excessive alerts may produce fatigue and be ignored. The next generation of electronic tools should therefore be non-disruptive when risk is low, actionable when risk is indeterminate or high, and linked to locally available services such as FibroScan appointment scheduling, ELF testing, MRI referral, or hepatology consultation. Predictive models based on structured electronic health record data may further improve risk stratification beyond simple fibrosis scores, particularly in populations with obesity and diabetes (61). Nevertheless, such models must be transparent, externally validated, and calibrated for local populations before being incorporated into clinical decision-making.

A second priority is multidisciplinary collaboration among endocrinology, hepatology, nutrition, radiology, nursing and, where available, obesity medicine. MASLD/MASH in T2DM is not a single-organ disease. Endocrinologists are well placed to identify at-risk patients and optimize metabolic therapy; hepatologists are essential for advanced fibrosis, cirrhosis, portal hypertension, hepatocellular carcinoma surveillance and liver-directed pharmacotherapy; dietitians and diabetes educators are central to lifestyle intervention; imaging units provide FibroScan, MRI-PDFF and MRE; and nursing teams can support education and follow-up adherence. Structured case-finding algorithms have increased access to specialty assessment for patients with diabetes and suspected advanced liver disease (62). Future models should formalize these roles and establish feedback loops between specialties.

This multidisciplinary structure is becoming more important as disease-specific therapies become available for noncirrhotic MASH with moderate to advanced fibrosis (52, 57). The previous argument that fibrosis detection had limited practical value because treatment options were few is becoming less persuasive. Patients with significant fibrosis may now require more precise staging, specialist assessment, eligibility evaluation for liver-directed therapy and long-term surveillance. Endocrinology clinics should serve as the first detection point, but high-risk disease should be managed through coordinated pathways rather than isolated specialty silos.

A third priority is local validation of FIB-4 and FibroScan thresholds in Chinese patients with T2DM. Most widely used cut-offs were developed and validated in mixed or Western cohorts, and performance may differ by age, ethnicity, body composition, diabetes status, obesity pattern and background liver disease prevalence. This issue is especially relevant in Chinese and other Asian populations, where body mass index may underestimate visceral adiposity and metabolic risk. Studies in Chinese patients with T2DM and NAFLD have linked liver fibrosis with age, systolic blood pressure, ALT, GGT, platelet count and other metabolic variables, supporting the need for population-specific risk interpretation (63). Chinese T2DM cohorts also suggest that MAFLD/MASLD is common and that many patients fall into indeterminate or high-risk categories when conventional non-invasive scores are applied (64).

Local validation should refine, rather than invalidate, the proposed workflow. The conventional FIB-4 threshold of 1.3 and high-risk threshold of 2.67 can be used as provisional triage thresholds because they are recommended by current guidance, but prospective studies are needed to determine whether they optimally exclude or detect advanced fibrosis in Chinese T2DM patients. Similar calibration is needed for age-adjusted FIB-4 thresholds, LSM cut-offs for significant fibrosis, advanced fibrosis and compensated advanced chronic liver disease, CAP interpretation in central obesity, probe selection and management of discordant FIB-4 and FibroScan results. The 2024 Chinese guideline provides a national framework for MASLD screening and management (12), and subsequent commentary has highlighted the importance of integrating FIB-4 and transient elastography into Chinese practice (65).

Fourth, real-world studies and prospective cohort validation are needed. A proposed pathway should not be judged only by its consistency with guidelines. It should be tested in outpatient settings, where patient flow, appointment availability, insurance coverage, clinician behaviour and follow-up adherence shape outcomes. A prospective study could enroll consecutive T2DM patients in endocrinology clinics, automatically calculate FIB-4, direct indeterminate- and high-risk patients to FibroScan or ELF testing, refer high-risk patients to hepatology, and reassess patients at 24 and 48 weeks. Such a design could measure screening coverage, risk distribution, second-line testing completion, referral completion, changes in metabolic and liver-related indicators and loss to follow-up. Longer follow-up could evaluate progression to advanced fibrosis, cirrhosis, hepatocellular carcinoma, cardiovascular events and kidney outcomes.

Real-world validation should also compare different pathway designs. For example, one model may apply FIB-4 to all patients with T2DM, whereas another may prioritize patients with obesity, abnormal GGT, long diabetes duration, or multiple metabolic risk factors. One pathway may use FibroScan as the default second-line test, whereas another may use ELF where elastography is unavailable. Some hospitals may adopt direct hepatology referral for FIB-4 >2.67, while others may require LSM confirmation before referral. These variations are not weaknesses; they are implementation questions. Comparative real-world studies can clarify which strategy provides the best balance between sensitivity, referral burden, cost, and patient adherence.

The fifth priority is to convert the pathway into a hospital-level quality improvement project. MASLD/MASH screening should not depend solely on individual physician interest. It should be incorporated into institutional workflows, similar to diabetic retinopathy screening, nephropathy monitoring and cardiovascular risk assessment. Quality indicators may include the proportion of eligible T2DM patients with FIB-4 automatically calculated, the proportion of indeterminate- or high-risk patients completing second-line testing, the proportion of high-risk patients completing hepatology referral and the proportion reassessed at 24 or 48 weeks. Implementation science frameworks such as RE-AIM and standard implementation outcome measures can help evaluate reach, adoption, feasibility, fidelity and sustainability (66, 67). In this way, the pathway becomes a measurable quality target rather than a general recommendation.

For hospitals with limited access to FibroScan or MRI-PDFF, quality improvement should focus on staged implementation. The first stage may be FIB-4 automation and clinician education. The second stage may be building a referral channel for FibroScan or ELF testing. The third stage may involve hepatology co-management for high-risk patients. The fourth stage may incorporate MRI-PDFF or MRE for selected patients, clinical research, or treatment-response evaluation. This staged model is more realistic than requiring all hospitals to implement a complete pathway immediately. It also allows resource-limited centres to begin with low-cost fibrosis risk stratification while gradually expanding diagnostic capacity.

Several unresolved issues require further study. The optimal reassessment interval for low-risk T2DM patients remains uncertain, especially in those with obesity, worsening glycaemic control or long diabetes duration. The clinical meaning of small changes in CAP or LSM over 24 to 48 weeks also needs clarification. The impact of incretin-based therapy, SGLT2 inhibitors and liver-directed therapy on pathway-level outcomes has not been fully defined. Future studies should also evaluate patient-reported outcomes, health literacy, cost-effectiveness and equity. A pathway that works only in tertiary hospitals or among highly adherent patients would have limited public health value.

In summary, future development should move from conceptual pathways to validated systems. Electronic FIB-4 automation can improve detection; multidisciplinary collaboration can connect metabolic treatment with liver-specific care; local validation can adapt thresholds to Chinese T2DM populations; prospective cohorts can test feasibility and outcomes; and quality improvement programmes can make MASLD/MASH screening part of routine diabetes care. The long-term goal is not simply to screen more patients, but to identify those at meaningful risk, intervene earlier, reduce unnecessary referrals and build a sustainable model of hepatic risk management within endocrinology practice.

## Limitations of this review

Several limitations should be acknowledged. This is a narrative review rather than a systematic review, and the included evidence spans different terminologies, populations, assessment methods and healthcare settings. Many pivotal studies were conducted under the NAFLD/NASH nomenclature, whereas current clinical practice is moving toward MASLD/MASH. In addition, proposed thresholds for FIB-4, VCTE, ELF, MRI-PDFF and MRE may not perform uniformly across age groups, ethnic populations, obesity phenotypes and diabetes durations. The pathway proposed here should therefore be regarded as a clinically informed framework that requires prospective validation, local calibration and resource-sensitive implementation before broad adoption.

## Conclusion

MASLD/MASH has become an important but still insufficiently integrated component of chronic disease management in patients with T2DM. Current evidence and guidelines indicate that this population carries a high burden of steatosis, MASH and clinically significant fibrosis, and that liver-related risk cannot be reliably excluded by aminotransferase levels or incidental imaging alone (2, 3, 6, 10–13, 24, 25). For endocrinology outpatient clinics, the priority should therefore shift from passive recognition of fatty liver to proactive identification of advanced fibrosis and at-risk MASH.

At the same time, the pathway should be applied with appropriate caution. FIB-4 is a useful but imperfect entry test; age-adjusted interpretation is needed in older adults; and ELF, FibroScan, MRE or specialist evaluation may be selected according to whether the immediate objective is referral triage or fibrosis staging. The proposed 24-week and 48-week checkpoints, role allocation and quality indicators are pragmatic implementation choices rather than fixed guideline mandates. Prospective validation in different healthcare systems, including Chinese endocrinology settings, is required before any single version of the pathway can be considered definitive. The pathway should also incorporate alcohol history, possible MetALD, normal-ALT fibrosis, and extrahepatic cancer screening needs into routine risk review rather than treating these issues as outside diabetes care.

A pathway based on automated FIB-4 calculation, second-line FibroScan CAP/LSM or ELF testing, high-risk referral, metabolic intervention and structured follow-up provides a feasible framework for this shift. It is not intended to replace hepatology assessment or histological diagnosis in selected patients. Rather, it offers an operational triage model that helps endocrinologists identify low-risk patients suitable for longitudinal metabolic monitoring, intermediate-risk patients requiring further non-invasive testing and high-risk patients requiring specialist hepatology evaluation. This structure is aligned with current ADA, AGA, EASL-EASD-EASO, AASLD, AACE and Chinese guidance, while adapting those recommendations to the workflow of diabetes clinics (10–13, 24, 25).

The value of the pathway should be judged by implementation and outcomes, not by conceptual completeness. Screening coverage, FIB-4 automation, FibroScan or ELF completion, MRI-PDFF or MRE use in selected patients, referral completion, follow-up adherence and longitudinal changes in metabolic and liver-related indicators should all be evaluated. Evidence from automated fibrosis score calculation, electronic reminders and structured case-finding suggests that the effectiveness of MASLD/MASH screening depends not only on test performance, but also on whether patients move reliably from risk identification to follow-up and care (36, 59, 62, 68).

Pathway evaluation should also document whether high-risk patients receive appropriate assessment of alcohol exposure, counselling for alcohol reduction when MetALD is possible, age- and sex-appropriate extrahepatic cancer screening, and HCC surveillance when advanced fibrosis or cirrhosis is confirmed or strongly suspected.

Future work should focus on prospective and real-world validation of endocrinology-based MASLD/MASH pathways, particularly in Chinese and broader Asian T2DM populations. Local studies are needed to clarify FIB-4 and FibroScan thresholds, define referral criteria, evaluate adherence and determine whether pathway implementation improves detection of advanced fibrosis without creating excessive referral burden (63–65). In parallel, hospital-level quality improvement projects should incorporate MASLD/MASH screening into routine diabetes care in the same way that retinopathy, nephropathy, cardiovascular risk, neuropathy and foot complications are systematically assessed.

In summary, endocrinology outpatient clinics are a key entry point for early recognition and long-term management of MASLD/MASH in patients with T2DM. The contribution of this review is an implementation-oriented synthesis rather than a new diagnostic threshold: it reframes MASLD/MASH as an under-integrated component of diabetes complication care, adapts multisociety guidance into a practical outpatient workflow, and proposes measurable indicators for real-world quality improvement. A closed-loop approach that begins with FIB-4-based risk stratification, proceeds through FibroScan- or ELF-based refinement and high-risk referral, and returns to structured follow-up may help move care from incidental detection of fatty liver toward prevention of advanced fibrosis and systemic metabolic complications.

## Glossary

AACE: American Association of Clinical Endocrinology
AASLD: American Association for the Study of Liver Diseases
ADA: American Diabetes Association
AGA: American Gastroenterological Association
ALT: alanine aminotransferase
APRI: AST-to-platelet ratio index
AST: aspartate aminotransferase
CAP: controlled attenuation parameter
CKD: chronic kidney disease
ELF: enhanced liver fibrosis test
FIB-4: fibrosis-4 index
GLP-1RA: glucagon-like peptide-1 receptor agonist
GGT: gamma-glutamyl transferase
HCC: hepatocellular carcinoma
LSM: liver stiffness measurement
MASLD: metabolic dysfunction-associated steatotic liver disease
MASH: metabolic dysfunction-associated steatohepatitis
MRE: magnetic resonance elastography
MRI-PDFF: magnetic resonance imaging-proton density fat fraction
NAFLD: non-alcoholic fatty liver disease
NASH: non-alcoholic steatohepatitis
NFS: NAFLD fibrosis score
NIT: non-invasive test
SGLT2i: sodium-glucose cotransporter 2 inhibitor
T2DM: type 2 diabetes mellitus
VCTE: vibration-controlled transient elastography
AUD: alcohol use disorder
MetALD: metabolic dysfunction and alcohol-associated liver disease.
